# Non-Coding RNAs as Potential Neuroprotectants against Ischemic Brain Injury

**DOI:** 10.3390/brainsci3010360

**Published:** 2013-03-20

**Authors:** Prameet Kaur, Fujia Liu, Jun Rong Tan, Kai Ying Lim, Sugunavathi Sepramaniam, Dwi Setyowati Karolina, Arunmozhiarasi Armugam, Kandiah Jeyaseelan

**Affiliations:** 1 Department of Biochemistry and Neuroscience Research Centre, Centre for Translational Medicine, Yong Loo Lin School of Medicine, National University of Singapore, 14 Medical Drive, Singapore 117599, Singapore; E-Mails: a0030101@nus.edu.sg (P.K.); a0066366@nus.edu.sg (F.L.); a0030915@nus.edu.sg (J.R.T.); bchlimky@nus.edu.sg (K.Y.L.); bchss@nus.edu.sg (S.S.); bchkds@nus.edu.sg (D.S.K.); bchaa@nus.edu.sg (A.A.); 2 Department of Anatomy and Developmental Biology, School of Biomedical Sciences, Faculty of Medicine, Nursing and Health Sciences, Monash University, Clayton, Victoria 3800, Australia

**Keywords:** non-coding RNAs, neuroprotectant, ischemia, brain, miRNA, lncRNA, piRNA

## Abstract

Over the past decade, scientific discoveries have highlighted new roles for a unique class of non-coding RNAs. Transcribed from the genome, these non-coding RNAs have been implicated in determining the biological complexity seen in mammals by acting as transcriptional and translational regulators. Non-coding RNAs, which can be sub-classified into long non-coding RNAs, microRNAs, PIWI-interacting RNAs and several others, are widely expressed in the nervous system with roles in neurogenesis, development and maintenance of the neuronal phenotype. Perturbations of these non-coding transcripts have been observed in ischemic preconditioning as well as ischemic brain injury with characterization of the mechanisms by which they confer toxicity. Their dysregulation may also confer pathogenic conditions in neurovascular diseases. A better understanding of their expression patterns and functions has uncovered the potential use of these riboregulators as neuroprotectants to antagonize the detrimental molecular events taking place upon ischemic-reperfusion injury. In this review, we discuss the various roles of non-coding RNAs in brain development and their mechanisms of gene regulation in relation to ischemic brain injury. We will also address the future directions and open questions for identifying promising non-coding RNAs that could eventually serve as potential neuroprotectants against ischemic brain injury.

## 1. Introduction

The Encyclopedia of DNA Elements (ENCODE) project has recently determined that more than 74.7% of the entire human genome is transcribed into primary transcripts [1]. This is indeed remarkable given that only a mere 2.94% of the genome contributes to exons of protein-coding genes, thus emphasizing the prevalence of non-coding RNA (ncRNA) transcripts [2]. These non-protein coding transcripts were initially brushed away as “transcriptional noise” due to the lack of evidence of their functionality [3]. Recent studies have established that these RNA transcripts of various sizes are derived from different areas of the genome including untranslated regions as well as seemingly untranscribed regions and also within introns [3]. These are generally referred to as non-coding RNAs (ncRNAs) that display a broad range of effects on chromatin architecture, transcriptional regulation, posttranscriptional processing and translation [4,5]. 

Studies of this uncharacterized territory of the human genome are beginning to show functional importance in most processes, including regulation of the brain. ncRNAs were found to show brain specific expression and function [6,7,8]. These ncRNAs are regulated during neuronal development [9,10] and are also shown to be associated with neurological diseases [11,12]. Distinct temporal and spatial expression of specific ncRNAs has been observed during cerebral ischemia in *in vivo* models as well [13,14]. Ischemic stroke comprises of 87% of all stroke cases, with recombinant tissue plasminogen activator (rt-PA) being the only approved drug [15,16]. However, its use is limited due to the risk of cerebral haemorrhage and the narrow therapeutic window of 4.5 h [17]. Clinical trials using antithrombotic and anticoagulant agents to salvage or protect neuronal and non-neuronal cells have been proven unsuccessful [17]. Recent studies have shown that gene dysregulation leading to apoptotic events during ischemia could be attributed to the derailment of ncRNAs [18,19]. Several ncRNAs described to date have been shown to either cause cell death or protect neurons and non-neuronal cells from ischemic death [20,21,22]. Therefore, studies to characterize the roles of ncRNAs in the pathogenesis of ischemic injury are crucial to decipher the complex mechanisms at play. Furthermore, modulating their expression could potentially serve as an alternative therapeutic strategy. In-depth functional studies could therefore identify specific endogenous ncRNA-based regulators that can be modulated to impede dysregulation of gene expression associated with ischemic cell death [23]. This review will cover the biogenesis of ncRNAs, their characterized functions in neurogenesis as well as their roles upon dysregulation in ischemic preconditioning and disease.

## 2. Types and Biogenesis of ncRNAs Associated with Brain and Ischemia

ncRNAs comprise of broad range of transcripts, differing in size with various functions attached to each subtype. The 2 well-known subclasses of ncRNAs are the “housekeeping” ncRNAs (ribosomal RNA, transfer RNA, small nuclear RNA, small nucleolar RNA) and the recently characterized regulatory ncRNAs (e.g., microRNA, long ncRNA. See Table 1). Regulatory ncRNAs can be further classified based on their sizes as short (<200 bp) or long (>200 bp) ncRNAs. microRNAs (miRNAs) are the most well-characterized and widely-studied group of short ncRNAs in ischemic brain injury whereas the functions of the long non-coding RNAs (lncRNAs) and the piwi-interacting RNAs (piRNAs) are just beginning to be unraveled. 

**Table 1:** Classification and functional roles of non-coding RNA (ncRNA) in humans.

ncRNA	Function
*House-keeping ncRNAs*	
transfer RNA (tRNA)	mRNA translation [24]
ribosomal RNA (rRNA)	mRNA translation [24]
small nucleolar RNA (snoRNA)	rRNA modification [24]
small nuclear RNA (including spliceosomal RNA)	RNA splicing, polyadenylation [24]
*Regulatory ncRNAs: Short ncRNA (<200 nt)*	
microRNA (miRNA)	degradation of mRNA or repression of translation [25,26]
piwi-interacting RNA (piRNA)	regulation of transposon activity and chromatin state [27]
repeat-associated short interfering RNA (rasiRNA)	regulate germline transposition activity [28]
tRNA-derived RNAs	Translational repression [29]
Telomere small RNAs (tel-sRNAs)	Telomere maintenance [30]
Centrosome-associated RNAs (crasiRNAs)	Guide local chromatin modifications [31]
*Regulatory ncRNAs: Long ncRNA (>200 nt)*	
Intergenic ncRNA	Epigenetic regulators of transcription in *cis*/in *trans* [32]
Intronic ncRNA	Transcriptional, posttranscriptional regulation, precursors for small ncRNAs [33]
Antisense transcript	mRNA stability of its homologous coding gene [34]
Pseudogene transcript	Generation of natural antisense transcripts or competing endogenous RNAs, stabilization of its coding transcript by competitively binding miRNA [35,36]
Mitochondrial ncRNA (ncmtRNAs)	Cell cycle and proliferation by unknown mechanisms [37]
Repeat-associated ncRNA	Regulation of repeat silencing [38]
Satellite ncRNA	Involvement of formation and function of centromere-associate complexes [39]
Repetitive RNAs	Epigenetic regulation? Other mechanisms? [39]
Tiny transcription initiation RNAs (tiRNAs)	Chromatin modifications and protein recruitment for transcriptional initiation [40]
Promoter upstream transcripts (PROMPTS)	Chromatin changes [41]
Transcripts of unknown function (TUFs)	Stem cell differentiation [42]
*Regulatory ncRNAs: Diverse sizes*	
Promoter-associated RNAs (PARs)	Gene repression in *cis* via interacting with PRC2 [43]
Enhancer-like ncRNA (eRNA)	Activation of promoter activity by unknown mechanism [44]

### 2.1. miRNAs

miRNAs are highly conserved small (17–24 nucleotides) endogenous molecules that mediate post-transcriptional gene silencing of mRNA in a sequence-dependent manner [25,26]. They are vital regulators of gene expression for neuronal function associated with synaptic plasticity, neurogenesis and neurodegeneration [9,45,46]. A miRNA can regulate the expression of hundreds of genes simultaneously, and several miRNAs can regulate a single mRNA cooperatively [47]. Moreover, Mukherji *et al.* [48] have shown that miRNAs can act as both a switch and fine-tuner of gene expression. The switch is regulated by miRNAs to establish a threshold level of target mRNA to repress protein production. Fine-tuning is determined near this threshold where protein expression responds sensitively to target mRNA input. 

**Figure 1:**
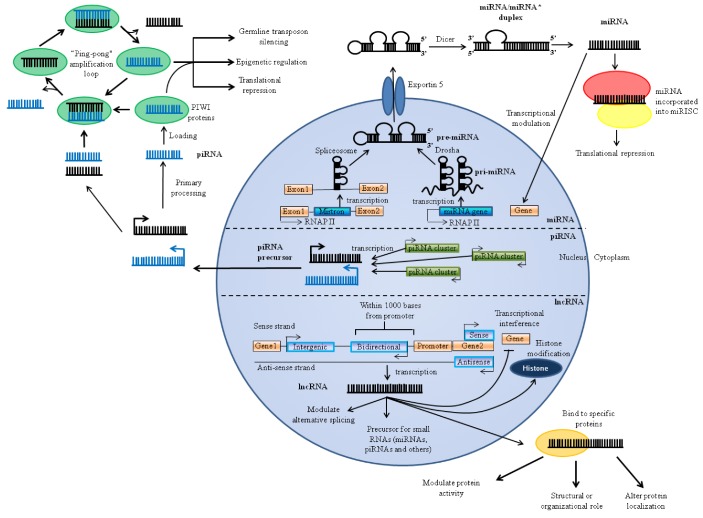
Biogenesis and function of the major ncRNAs (miRNAs, piRNAs, lncRNAs) implicated in ischemic injury.

Biogenesis of miRNAs is initiated by RNA polymerase II mediated transcription. miRNAs can exist as an independent gene or be located in introns of protein-coding genes (mirtrons) to give rise to primary miRNA transcripts (pri-miRNAs) (Figure 1). The folded pri-miRNA hairpins are cleaved by Drosha in the nucleus, exported into the cytoplasm and subsequently cleaved by Dicer to produce ~20 nt miRNA/miRNA* duplexes. The strand with a less thermodynamically stable 5′ end usually acts as the mature miRNA whereas the other strand is degraded. In certain cases, both strands are viable and become functional miRNAs. Thereafter, a mature miRNA is incorporated into a multiprotein complex known as the RNA-induced silencing complex (RISC), which also contains Argonaute proteins, to form miRISC. In the miRISC formation, miRNAs base pair to target mRNAs, generally on the 3′ untranslated region (UTR) and induce their translational repression or deadenylation and degradation [49]. Recent findings demonstrate that miRNAs are also capable of regulating gene expression at the transcriptional level [50,51,52]. 

### 2.2. lncRNAs

lncRNAs are RNA transcripts longer than 200 nucleotides that make up the largest portion of the transcriptome [53]. lncRNAs have been shown to play important roles in embryogenesis and development of the central nervous system [7]. They coordinate gene expression through epigenetic modification, mRNA splicing, control of transcription or translation and genomic imprinting which is determined by their structure and association with the gene loci [54]. lncRNAs are transcribed by RNA polymerase II, and some lncRNAs are processed in a similar manner to mRNAs, undergoing splicing, polyadenylation and 5′-capping [24,55]. lncRNAs originate from intronic, exonic, intergenic, promoter regions, 3′UTRs and 5′UTRs (Figure 1). Some lncRNAs can be transcribed as bidirectional transcripts (derived from divergent transcription within 1000 bp of a promoter) [56] and can also be derived from pseudogenes. A handful of lncRNAs are conserved [32] while other well-characterized functional mammalian lncRNAs, such as myocardial infarction associated transcript (Gomafu) [57], Dlx6 antisense RNA 1 (Evf-2) [58] and HOX transcript antisense RNA (HOTAIR) [59] exhibit poor sequence conservation across species. Nevertheless, lncRNAs have shown important implications in cellular functions [60].

### 2.3. piRNAs

piRNAs are small ncRNAs of 26–31 nucleotides (longer than miRNAs) which form complexes with Piwi proteins of the Argonaute family [61]. The majority of the mammalian piRNAs map uniquely in the genome and cluster to a small number of loci [62,63,64]. The primary role of these small RNAs has been shown to suppress transposon activity during germ line development by complementation to transposable and repetitive elements [27,65]. piRNAs are generated by two proposed pathways: the primary processing pathway and the “ping-pong” amplification loop. In the primary processing pathway, primary antisense transcripts are transcribed from transposons and/or piRNA clusters (genomic regions depleted in transposons) and processed into piRNAs by unknown mechanisms [66,67,68]. piRNAs derived from this mechanism provide an initial pool of piRNAs that target multiple transposable elements [68], with recently characterized functions in somatic cells and regulation of the cell cycle of mesenchymal stem cells [69,70]. This is followed by the second “ping-pong” amplification loop (Figure 1), which further increases the abundance of piRNAs and transposon silencing [27,62,67,71]. Single-stranded precursors transcribed from transposable elements and other repetitive elements give rise to antisense piRNAs, which are loaded onto associated PIWI-proteins. This complex recognizes and cleaves complementary transcripts, generating further sense piRNAs that exactly correspond to the original primary piRNA sequences and are loaded onto another PIWI protein for cleavage, giving rise to the “ping-pong” mechanism [62]. 

Many other classes of regulatory ncRNAs have been found to be associated with the different components of a gene (Table 1). Repeat-associated small interfering RNAs (rasiRNAs) [28], repeat-associated ncRNAs [38], transcription initiation RNAs (tiRNAs) [40], and promoter-associated RNAs (PARs) [43] are ncRNAs that are currently gaining attention. Although the functions of these recently discovered classes remains to be elucidated in neurogenesis and ischemic injury, they are postulated to be involved in transcriptional regulation [72]. 

## 3. ncRNAs in Brain Development and Ischemic Injury

Determining the role of ncRNAs in brain development is the first step in establishing their importance in proper brain function. Dysregulation of any part of this intricate process upon ischemic injury could account for disruption of brain function and hence damage. Moreover, ischemic preconditioning can confer resistance to subsequent lethal ischemic events. Most importantly, pathogenic events and processes that unfold upon ischemic insult provide the most appropriate model for studying the neurotoxic effects of this disease. We will cover the current research findings in these areas to provide insights into promising neuroprotective targets for modulation. 

### 3.1. ncRNAs in Brain Development

miRNAs have been reported to be crucial for neuronal development. Deletion of Dicer, in neural stem cells causes massive hypotrophy of the postnatal cortex, lethality, ablation in late embryonic stages in the central nervous system (CNS). In addition, it leads to dysfunction in migration of late-born neurons in the cortex and oligodendrocyte precursor expansion, as well as differentiation in the spinal cord [73,74]. Moreover, conditional Dicer deletion in forebrain and hippocampal neurons results in changes in dendrite morphology, spine length, apoptosis, microcephaly, ataxia, and lethality within three weeks after birth [75]. Studies on neuron enriched miRNAs have shed light on some mechanisms during neuronal development, which involve neuronal stem/progenitor cells proliferation and differentiation, neuritogenesis and outgrowth, synapse formation and plasticity.

Brain-enriched miR-124 is well-conserved from worms to humans and is estimated to be the most abundant miRNA in the brain [8]. miR-124 may contribute to maintaining neuronal identity by suppressing non-neuronal gene expression in neurons [76]. Furthermore, miR-124a-1 knockout mice showed severe consequences for neuronal survival and axonal outgrowth following reduction of miR-124 [77]. miR-124 is highly expressed in differentiating and mature neurons and exhibits increased expression during neuronal differentiation [78]. This process is controlled by transcription factor RE1-silencing transcription factor (*REST*), a negative regulator of miR-124 via repressor element (RE1) binding sites in the miR-124 genomic loci [79]. miR-124 is a critical switch for neural stem cell exit from multipotency and differentiation towards a neuronal phenotype by targeting small *C*-terminal domain phosphatase 1 (*SCP1*) [80], polypyrimidine tract binding protein 1 (*PTBP1*) [81] and SRY-box transcription factor (*Sox9*) [9]. Upon ischemic injury, expression of miR-124 is reduced in neuronal progenitor cells of the subventricular zone (SVZ). Downregulation of miR-124 upregulates jagged 1 (*Jag1*), a ligand of the Notch signaling pathway, which mediates neuronal progenitor cell (NPC) proliferation leading to stroke-induced neurogenesis [82]. Furthermore, miR-124 is also proposed to be a biomarker in cerebral ischemia [83]. 

miR-9 is another highly conserved, brain specific miRNA, with its expression largely confined to the nervous system [84,85]. Several studies suggest that miR-9/miR-9* suppresses neuronal progenitor proliferation and promotes neural differentiation via nuclear receptor subfamily 2, group E, member 1 (*Nr2e1*/*TLX*), *REST*, and REST corepressor 2 (*CoREST*) [86,87]. In contrast, in human embryonic stem cell-derived neural progenitors, miR-9 promotes proliferation and inhibits migration by targeting *stathmin 1* [88]. miR-9 has been shown to play an important role in telencephalic formation by either promoting or suppressing NPC proliferation through different targets at various stages of brain development [89]. In differentiated neurons, miR-9 controls axonal extension and branching by regulating translation of microtubule-associated protein 1B (*Map1b*), an important factor for microtubule stability [90]. Brain specific miR-128 is reported to promote neuronal differentiation by repressing nonsense-mediated decay to allow upregulation of tubulin, beta 3 class III (*Tuj-1*) and microtubule-associated protein 2 (*Map2*) mRNA levels and, downregulation of POU domain, class 5, transcription factor 1 (*Oct4*) mRNA level in P19 stem cells [91]. Both miR-9 and miR-128 are downregulated in SVZ following cerebral ischemia, indicating dysregulation of neuronal function, though their exact role in cerebral ischemia is still poorly understood [82].

Several other miRNAs have also been identified as either sharing the role in central nervous system development, or in the differentiation of individual cell types. Another brain specific miRNA, miR-134 was presented as a regulator of cortical development by regulating NPC proliferation, neuron migration, and embryonic neuronal maturation via its interaction with its targets doublecortin (*Dcx*) and chordin-like 1 (*Chrdl-1*) [92]. It also functions in modulating the size of dendritic spines-postsynaptic sites of excitatory synaptic transmission, by targeting the LIM-domain containing protein kinase (*Limk1*) [45], regulates sirtuin 1 (*SIRT1*)-mediated synaptic plasticity and memory formation [93] as well as dendritogenesis by targeting *Pumilio2* [94]. miR-134 is significantly upregulated upon cerebral ischemia indicating that injured cells could be actively involved in regeneration during the first 24 h of reperfusion [13]. miR-29b is significantly induced with neuronal maturation and functions as a neuron apoptosis inhibitor by targeting pro-apoptotic BH3-only family genes, BCL2-like 11 (apoptosis facilitator) (*Bim*), BCL2 modifying factor (*Bmf*), harakiri, BCL2 interacting protein (contains only BH3 domain) (*Hrk*), and BCL2 binding component 3 (*Puma*) [95]. Several other miRNAs such as miR-137 and miR-34a have been found to function in axon and dendrite development and synaptic plasticity (Table 2). miR-34a and -137 have also been reported to be dysregulated at 24 h in the rat stroke model (transient middle cerebral artery occlusion, MCAo) [13] indicating that processes for neurogenesis and maintenance of the neuronal phenotype are altered upon ischemic injury.

**Table 2:** List of miRNAs and lncRNAs involved in the different processes in ischemic injury.

miRNA	Target gene	Effect	lncRNA	Associated gene	Effect
*Brain development*					
miR-124	*SCP1*	Promotes neurogenesis [80]	*Anti-NOS2A*	*NOS2A*	Possibly downregulate NOS2A expression, regulate neuronal differentiation [96]
	*PTBP1*	Promotes neuronal differentiation [81]	*Neurogranin* and *Camk2n1* associated sense and antisense transcripts	*Neurogranin*, *Camk2n1*	Posttranscriptional regulation of differentiation [97]
	*BAF53a*	Promotes neuronal differentiation [98]	Tie-1AS	*Tie-1*	Regulation of vascular development [99]
	*SOX9*	Promotes neuronal differentiation [9]	Tsx	Unknown	Learning and behavior [100]
	*Jag1*	Promotes neural progenitor cells proliferation [82]	Malat-1	SR proteins	Synapse formation and/or maintenance [101,102]
miR-9*	*BAF53a*	Promotes neuronal differentiation [98]	FGF-AS	*FGF*	Downregulates proliferation of neural progenitor cells [103,104]
	*CoREST*	Promotes neuronal differentiation [86]	ANRIL	Unknown	Risk and recurrence of stroke [105]
	*TLX*	Promotes neuronal differentiation [87]	Evf2	*Dlx5/6*	GABAergic interneuron development [106]
	*STMN1*	Promotes neuronal differentiation [88]	Sox2ot	*Sox2*	Expressed concurrently with Sox2 [107]
miR-9	*REST*	Promotes neuronal differentiation [86]	asOct4-pg5	*Oct4*	Downregulates transcription of Oct4 resulting in differentiation [108]
miR-134	*Dcx*, *Chrdl-1*	Promotes neuronal differentiation [92]	BDNF-AS	BDNF	Downregulates transcription of BDNF and BDNF mRNA [109]
miR-29b	*Bim*, *Bmf*, *Hrk*, and *Puma*	Promotes neuronal differentiation [95]			
miR-137	*Mib1*	Dendritic morphogenesis, neuronal maturation, spine development [110]			
miR-34a	*SIRT1*	Promotes neuronal differentiation and neurite elongation [111]			
miR-132	*p250GAP*	Enhances dendritic morphogenesis [112]			
	*IL-6*, *CCL2*, *CCL20*, *TSLP*	Integration of newborn neurons into adult brain synaptic circuitry [113]			
miR-338	*COXIV*	Regulates axonal respiration and function [114]			
miR-26a	*MAP2*	Regulates synaptic plasticity [115]			
miR-125b	*NR2A* (NMDA receptor subunit)	Regulates spine morphology and synaptic plasticity [116]			
miR-138	*APT1*	Regulates size of dendritic spines [117]			
miR-138	*Lypla1*	Regulates synaptic plasticity and spine morphology [118]			
miR-219	*CaMKIIgamma*	Regulates fast neurotransmission and synaptic plasticity [119]			
miR-375	*HuD*	Regulates dendrite maintenance [120]			
*Ischemic preconditioning*					
miR-200b/c and miR-429	*PHD2*	Provides neuroprotection [121]			
miR-199a	*SIRT1*	Reduces ischemic tolerance [122]			
*Hypoxia*					
miR-199a-5p	*HIF-1a*	Inhibits apoptosis [123]	5′aHIF-1α	*HIF-1α*	Prevents export of HIF-1α mRNA into cytoplasm [124]
miR-17-92 cluster	*HIF-1a*	Inhibits cancer cells proliferation [125]	3′aHIF-1α	*HIF-1α*	Downregulates HIF-1α mRNA [126]
miR-155	*HIF-1a*	Inhibit hypoxia [127]	aHig-1	*Hig-1*	Inhibits translation of Hig-1 [128]
miR-138	*HIF-1a*	Inhibits apoptosis and migration [129]	H19	IGF-2	Induced upon hypoxia, regulates expression of IGF-2, precursor for miR-675 [130,131]
miR-107	*HIF-1β*	Inhibits differentiation [132]	PTENP1	PTEN	Sequesters miRNAs acting on PTEN mRNA, cell death [133]
miR-20b	*VEGFA*	Inhibits tumor growth [134]			
miR-15a	*VEGFA*	Inhibits angiogenesis [135]			
miR-16	*VEGFA*	Inhibits angiogenesis [135]			
miR-519c	*HIF-1a*	Promotes angiogenesis [136]			
miR-93	*VEGFA*	Inhibits angiogenesis [137]			
miR-126	*VEGFA*	Inhibits angiogenesis [138]			
miR-200a	*Flt-1*	Inhibits tumour invasion [139]			
miR-145	*BNIP3*	Inhibit apoptosis [140]			
miR-221/222	*PUMA*	Inhibits apoptosis [141]			
*Excitotoxicity*					
miR-223	*NR2B*, *GluR2*	Inhibits excitotoxicity [142]	*CCND1* promoter associated lncRNA	*CCND1*	Represses CCND1 expression, cell survival [143]
miR-181a	*GluA2*	Inhibits excitotoxicity [144]			
*Inflammation*					
miR-146a/b	*IRAK1*, *TRAF6*	Inhibits inflammation [145]	17A	*GPR51*	Induced upon inflammation [146]
miR-146a	*TLR4*	Inhibits inflammation [147]			
miR-181c	*TNF-α*	Inhibits inflammation [148]			
miR-125b	*TNF-α*	Inhibits inflammation [149]			
miR-17	*ICAM1*	Inhibits recruitment of immune cells [150]			
miR-126	*VCAM1*	Inhibits recruitment of immune cells [151]			
miR-130a	*AQP4*	Reduces edema [52]			
miR-320a	*AQP4*	Reduces edema [22]			
*Oxidative stress*					
miR-145	*SOD2*	Inhibits anti-oxidant defense [152]	MSUR1	Unknown	Reduces ROS and oxidative damage [153]
miR-101	*COX2*	Reduces ROS production [154]	Gadd7	Unknown	Induced upon oxidative stress, cell death [155]
*Apoptosis*					
miR-15a	*BCL2*	Promotes cell death [156]	TUG1	Cell cycle genes	Induced by p53 upon DNA damage, cell death [157]
miR-29b	*BCL2L2*	Promotes cell death [158]			
miR-497	*BCL2*, *BCL2L2*	Promotes cell death [21]			
miR-21	*FASLG*	Inhibits cell death [20]			

Similarly, ischemic insult can also give rise to dysregulation of lncRNAs essential to neurogenesis, affecting the neuronal machinery, thereby conferring toxicity to the cells [10,159]. Inducible nitric oxide synthase (*NOS2A*) is upregulated during neurogenesis in the mammalian brain. This profile was observed to be opposite to that of an ncRNA antisense to the gene (*anti-NOS2A* RNA) during differentiation of human embryonic stem cells. This suggests involvement of human anti-NOS2A RNA in regulation of neuronal differentiation by suppressing NOS2A gene expression [96]. The expression of *NOS2A* is also induced after focal cerebral ischemia to stimulate neurogenesis in the adult rat dentate gyrus [160], but the expression of *anti-NOS2A* in this condition is unknown. Determination of expression of the ncRNA can pave the way for possible modulation of this neuroprotective enzyme. 

Brain-derived neurotrophic factor (BDNF) is a crucial determinant of neuronal outgrowth and survival. A conserved transcript antisense to BDNF has recently been characterized to negatively modulate transcription as well as translation of the *BDNF* mRNA [109]. Targeting the BDNF-AS lncRNA to favour expression of *BDNF* mRNA upon ischemia induced apoptosis could thus prove to be a potential neuroprotectant. Likewise, several sense and antisense transcripts are also actively expressed from the Neurogranin (*Nrgn*) and calcium/calmodulin-dependent protein kinase II inhibitor 1 (*Camk2n1*) loci during cerebral corticogenesis. This increases the diversity of posttranscriptional regulation, resulting in possible cell- and time-specific regulation [97]. Up-regulation of neurogranin mRNA and protein expression upon treatment with retinoic acid post-MCAo also suggests a neuroprotective role that can be modulated by ncRNAs [161]. Likewise, the expression of *Camk2n1* is downregulated upon acute spinal cord injury [162], further supporting the need to determine the functions of its associated lncRNAs for their modulation as potential neuroprotectants. 

Expression of another natural antisense transcript for tyrosine kinase containing immunoglobulin and epidermal growth factor homology domain-1 (tie-1), tie-1AS lncRNA has been detected in the brain of embryonic zebrafish. The lncRNA selectively binds tie-1 mRNA *in vivo* and regulates tie-1 transcript levels, resulting in specific defects in endothelial cell contact junctions *in vivo* and *in vitro*. This suggests transcriptional regulation of gene expression in vascular development and therefore, possibly in ischemic disease, which is characterized by vascular dysfunction as well [99].

The testis specific X-linked gene (*Tsx*) ncRNA is highly expressed in the brain and its deletion results in less fearful mice with enhanced hippocampal short-term memory, implicating a possible role in learning and behavior in mammals [100]. Metastasis-associated lung adenocarcinoma transcript 1 (*Malat1*) regulates synapse formation by modulating the expression of genes involved in synapse formation and/or maintenance [101]. Fibroblast growth factor-2 (*FGF-2*) upregulation during brain development is negatively regulated by an lncRNA antisense to its 3′UTR (*FGF-AS*) [103,104]. Ischemic brain injury also induces upregulation of the *FGF-2* transcript to promote proliferation of neural progenitor cells, though the expression of *FGF-AS* is not known [163]. The expression profile of another antisense lncRNA, cyclin-dependent kinase inhibitor 2A (*ANRIL*), is associated with risk and recurrence of stroke risk [105]. The ultraconserved (100% conserved across humans, mice, and rats) lncRNA, Evf-2 is critical for early GABAergic inter-neuron development as well as subsequent GABA-dependent connectivity in the adult brain [106]. 

LncRNAs regulating pluripotency associated factors (SRY-box containing gene 2 (Sox2) [107] and Oct4 [108]), corticogenesis regulating genes (SRY-box containing gene 4 (Sox4) and SRY-box containing gene 11 (Sox11) [164]), transcription factors (zinc finger homeobox 2 (zfh-5) [165] and ELK3, member of ETS oncogene family (ELK3) [166]) and neuronal and oligodendrocyte development have been identified in the brain [10]. Moreover, lncRNAs have been implicated in the modulation of mouse embryonic stem cell (mESC) pluripotency and are also established to be directly controlled by key mESC transcription factors [167].

Furthermore, Rajasethupathy *et al*. [168] demonstrated the presence of neuron-specific piRNAs in *Aplysia* (sea slug) with unique biogenesis patterns, nuclear localization and sensitivity to the serotonin neurotransmitter that is important for memory. The piRNA/piwi complex was also found to regulate the promoter of the transcription factor, activating transcription factor 4 (*CREB2*), by DNA methylation in an activity dependent manner. This may be an important and general mechanism of small RNA-mediated long-lasting regulation of gene expression in neurons that contributes to long-term memory. 

As identified above, several ncRNAs have displayed essential roles in neuronal development and ischemic-reperfusion injury. Many more miRNAs, lncRNAs and piRNAs, on the other hand, are characterized and their roles in neuronal functions are being reported. However, their expression under ischemic condition needs to be determined (Table 2). It is highly probable that their dysregulation leads to the progression of ischemic injury. Given their emerging importance in neurogenesis and neuronal function, dysregulation of ncRNAs essential to brain function following ischemic injury warrants further study as promising neuroprotectants.

### 3.2. ncRNAs in Ischemic Preconditioning

In addition to brain development, ischemic preconditioning can serve as another model to identify neuroprotective ncRNAs for ischemic stroke. Preconditioning of tissues with sub-lethal stresses or stimuli can result in resistance to subsequent lethal ischemic events. This phenomenon is known as ischemic tolerance. The concept of ischemic preconditioning (IP) was first described in ischemic hearts by Murry *et al*. [169] in 1986. A variety of stress factors can induce neuronal ischemic tolerance, including brief periods of ischemia [170], hypoxia, hypothermia, hyperthermia and chemicals. The phenomenon of IP for acute ischemic-reperfusion injury has been reproduced in various organs including the brain [171]. The concept of cerebral ischemic tolerance was first reported in the 1990s by Kitagawa *et al.* [170]. The team found that exposure to 2 or 5 min of transient ischemia, 24 or 48 h prior to 10 min global cerebral ischemia in gerbils was neuroprotective against neuronal cell death.

It has been known that brain ischemic tolerance occurs in two phases: an early phase that occurs several minutes or hours after preconditioning, and a late phase that takes place several days later. Rapid and delayed preconditioning in both the heart and brain acts via different mechanisms. Generally, early preconditioning is related to a rapid response such as changes in ion channel permeability and post-translational modifications of proteins, while late preconditioning involves gene activation and protein synthesis [172,173,174,175]. 

Changes in miRNA profiles have been observed following IP. Dharap *et al*. [176] profiled cerebral miRNAs in the cerebral cortex of rats subjected to 10 min of MCAo. They reported fifty one miRNAs displaying altered expression with fold change >1.5 at 6 h following IP. Of these, twenty miRNAs (miR-374, -98, -340-5p, -21, -352, -379*, -335, -181b, -26b, -15b, -146a, -466c, -292-5p, -328, -873, -494, -7d*, -345-5p, -30c-2*, -322*) maintained the changed level until 3 days after IP. Moreover, the authors indicated that MAP-kinase, mTOR signaling, Wnt and GnRH signaling pathways might be crucial during IP. Lee *et al*. [121] found two miRNA families, miR-200 (miR-200a, miR-200b, miR-200c, miR-141, miR-429) and miR-182 (miR-182, miR-183, miR-96), are selectively upregulated at 3 h after IP (15 min MCAo). The authors demonstrated that the miR-200 family increased neuronal cell survival upon *in vitro* ischemic insult (oxygen glucose deprivation, OGD) by targeting Prolyl-4-hydroxylase (*PHD2*) mRNA. Furthermore, miR-199a that targets *SIRT1* was reported [122] to be downregulated during 3-nitropropionic acid (3-NPA)-induced preconditioning in rat brain. miR-132 was also involved in preconditioning by targeting methyl CpG binding protein 2 (*MeCP2*) [177]. The role of lncRNAs during ischemic preconditioning, however, has yet to be determined. As a regulator of cellular functions just like miRNAs, a more in-depth elucidation of lncRNA function could likewise expand the scope of potential therapeutic targets. 

Reversal of neurogenic processes and dysregulation of ncRNAs upon IP indicates only a subset of ncRNAs that are altered upon ischemic injury. The direct impact on ncRNA expression upon ischemic-reperfusion injury can only be determined in appropriate models replicating the various pathological processes during ischemic injury, resulting in cell death.

### 3.3. ncRNAs in Ischemic Injury

Neuronal cell death is the main effect brought about by ischemic brain injury and the underlying cause for manifestation of impaired cerebral function. Neuronal cell death is the end-point resulting from a multitude of molecular events and processes that occur upon ischemia. These molecular processes have been described as an ischemic cascade [17]. However, it is inapt to describe the processes in the form of a cascade as some of these processes loop back to potentiate repeated activation of certain pathways and processes. Generally, there are a handful of key processes including hypoxia, oxidative stress, inflammation, edema formation and excitotoxicity that lead to the demise of neurons during cerebral ischemia [178]. Several neuroprotectants have been identified throughout these years, however their efficiency in regulating the above-mentioned processes has failed to show significant effects in clinical trials [17]. 

In 2008, Jeyaseelan *et al*. [13] demonstrated that miRNAs showed differential expression in brain and blood of the rat stroke model, MCAo. Furthermore, dysregulation of circulating miRNAs in young stroke patients has also been described by this same group [179]. This discovery highlighted the possibility of a novel class of potential neuroprotectants involved in cerebral ischemia. Thereafter, several studies [20,21,22] reported modulating miRNAs during cerebral ischemia could protect neurons from ischemic injury. 

On the other hand, Dharap *et al*. [14] reported the dysregulation of lncRNAs in rats subjected to focal ischemia. The group identified lncRNAs which showed >90% sequence homology with exons of protein-coding genes. The authors reported that the stroke-responsive lncRNAs were homologous to protein-coding genes involved in ribosomal complex formation, splicing, translation initiation, and nuclear import of mRNAs, possibly stabilizing those mRNAs to restore the protein synthesis inhibited during the acute phase after stroke. The stroke-responsive lncRNAs might also control chromatin modifications, transcription factor activity, and apoptosis [157,180,181]. Furthermore, Kalkkila *et al.* [182] reported the induction of short interspersed elements (*SINEs*) *B1* and *B2* in the CA1 region of the hippocampus upon global ischemia in Mongolian gerbils. This signifies that ncRNAs are stress-inducible factors in the central nervous system. Further studies are needed to characterize the importance of lncRNAs to post-stroke functional outcome. 

Similarly, Dharap *et al*. [183], also reported the dysregulation of piRNAs in ischemic injury. However, piRNAs that mediate the neuronal damage have not been identified. The mechanisms of piRNA function are not yet known but studies showing high expression of the human homolog of the Drosophila piwi, hiwi, in testis, kidney and brain suggest the importance of these piRNAs for normal functioning of all organs including the brain [184]. 

This part of the review will provide a comprehensive summary of ncRNAs, mainly miRNAs and lncRNAs, implicated in different segments of the ischemic cascade as well as to highlight their potential as neuroprotectants (Table 2).

#### 3.3.1. Hypoxia

Hypoxia, or deprivation of adequate oxygen supply, induces bioenergetic failure that is regarded as the main trigger of downstream cerebral ischemic cascades that comprise of excitotoxicity, oxidative stress, inflammation and apoptosis. These processes result in neuronal injury and death within hours of ischemia onset. Hypoxia inducible factor-1 (HIF-1), a basic helix-loop-helix (HLH) heterodimer, is the master transcriptional factor in response to hypoxia in various diseases [185]. This oxygen-regulated HIF-1α subunit dimerizes with the constitutively expressed subunit HIF-1β to form the HIF-1 protein. HIF-1 in turn regulates its downstream genes, which involves those that promote cell survival, (glucose metabolism, angiogenesis, erythropoiesis) and those that confer cell death (apoptosis) [186,187,188]. HIF-1 functions as a double-edged sword in ischemic stroke with both neuroprotective and detrimental effects, which are determined by the severity and duration of cerebral ischemia and cell type. During low-oxygen conditions, this transcription factor mediates an endogenous adaptive mechanism to maintain oxygen homeostasis [189]. However, failure to adapt to these low-oxygen conditions under chronic hypoxia will eventually lead to cell death via apoptosis, as in the case of ischemic stroke [190,191]. Moreover, the HIF-1α subunit shows biphasic activation in rat stroke models resulting in expression of apoptosis-related genes during the early chronic phase (8 h after stroke onset) but shifts to protective gene expression during the recovery phase (after 48 h) of cerebral ischemia [192]. 

miRNAs have been involved in hypoxia regulation by targeting hypoxia-induced mRNAs [193] and these miRNAs may also be crucial regulators during cerebral ischemia. miR-210, a hypoxia-induced miRNA, is regulated by *HIF-1α* [194]. miR-210 has been proven to function in ischemic stroke by inducing the Notch signaling pathway and indicated to be a clinical biomarker for ischemic stroke diagnosis and prognosis [195,196]. Other hypoxia-induced genes have also been identified with miRNAs validated to target these (Table 2). 

Furthermore, *HIF-1α* associated lncRNAs have also been identified. Two antisense transcripts (5′aHIF-1α, 3′aHIF-1α) conserved in humans and rodents [197], have been reported to be associated with the *HIF-1α* gene at the 5′ and 3′ ends respectively [124]. Both transcripts are localized to the nucleus and activated upon different stresses. In cancer cells, 3′aHIF-1α is upregulated upon hypoxia to downregulate the *HIF-1α* mRNA by binding to the 3′UTR [126]. Furthermore, 5′aHIF-1α accumulation at the nuclear membrane may have a role in decreasing mRNA levels by affecting the export of mRNAs into the cytoplasm. The two antisense transcripts might, alternatively, be involved in mRNA degradation or chromatin inactivation of the *HIF-1α* gene locus, along with posttranscriptional modulation by miRNAs [124]. 

Hypoxia-induced gene 1 (*Hig-1*), another gene related to differentiation and cell death/survival balance is induced in neuron-enriched primary cultures upon exposure to hypoxia [198]. *Hig-1* is temporally regulated during spinal cord development with the mRNA expression remaining high throughout the postnatal period. Nevertheless, the increased distribution of Hig-1 protein is observed to be switching from neuronal to glial cells during the development of the rat spinal cord. This occurs with high expression of an antisense transcript (aHig-1) in neurons, suggesting RNA degradation or inhibition of translation by Hig-1 resulting in the absence of the protein [128]. Functional characterization of this antisense lncRNA would be useful in elucidating its implication in ischemic injury. Another hypoxia-induced lncRNA, H19 fetal liver mRNA (*H19*), is expressed in fetal brain but is drastically reduced in adult brain [130]. H19 can also act as a precursor transcript for miR-675 in tumor cells [131]. Nonetheless, no reports regarding its significance in the ischemic brain have emerged. In addition, tumor suppressor *PTEN* (phosphatase and tensin homolog deleted on chromosome 10) is a negative regulator of neuronal cell survival. PTEN is upregulated upon ischemic injury [199,200] and attenuates hypoxia-mediated HIF-1α stabilization in glioblastoma [201]. The *PTEN* pseudogene (*PTENP1*) acts as a decoy for miRNAs that target the *PTEN* mRNA. Therefore, regulation of the *PTEN* mRNA is likely to regulate signaling downstream of HIF-1α in stroke indirectly [133].

#### 3.3.2. Excitotoxicity

During cerebral ischemia, the deprivation of oxygen and glucose leads to energy failure characterized by an impaired Na^+^/K^+^ ATPase pump [202]. This results in depolarization of neurons, releasing neurotransmitters (especially glutamate) into the synaptic cleft. Glutamate activates the different glutamate receptors, α-amino-3-hydroxy-5-methyl-4-isoxazolepropionic acid receptor (AMPA), metabotropic glutamate receptors (mGluR) and *N*-methyl-d-aspartate (NMDA) receptors [203,204,205], resulting in calcium (Ca^2+^) influx into the neurons and also the release of intracellular Ca^2+^ store [203,206]. This Ca^2+^ overload activates detrimental enzymes (endonucleases, proteases, lipase) [17,178,202], damages the mitochondria that causes the release of pro-apoptotic factors and generates reactive oxygen species (ROS) [207]. In addition, Ca^2+^ influx from NMDA receptors activates nitric oxide synthase (NOS) through the binding of calmodulin (cofactor for NOS) [208] and also activates phospholipase A_2_ which breaks down membrane phospholipids [209]. All of these processes contribute to an increase in oxidative stress thus resulting in cell death.

miR-223 has been demonstrated to target the glutamate receptor, ionotropic, *N*-methyl-d-aspartate 2B (*NR2B*) subunit of the NMDA receptor and glutamate receptor, ionotropic, AMPA2 (*GluR2*) subunit of the AMPA receptor [142]. miR-223 overexpression has been shown to be neuroprotective by preventing Ca^2+^ influx. Hence, delivery of miR-223 mimics can protect neurons from excitotoxic cell death. The *GluA2*/*GluR2* subunit of AMPA receptor has been shown to be the target of miR-181a. Thus, increasing miR-181a may prove to be neuroprotective [144]. Overexpression of brain-derived neurotrophic factor (*BDNF*) during cerebral ischemia induces expression of miR-132, which in turn increases the expression of NMDA receptor (*NR2A* and *NR2B* subunits) and mGluR (glutamate receptor, ionotropic, AMPA1 (alpha 1), *GluR1*) [210,211]. Hence, the use of miR-132 antagomir in this case, may have neuroprotective effects by suppressing glutamate receptor expression, thereby reducing excitotoxicity. 

Cyclin D1 (*CCND1*), a cell cycle related gene, is a critical mediator of ischemic neuronal cell death induced by excitotoxic NMDA receptors [143,212,213]. An lncRNA, transcribed from the promoter region of the gene *CCND1*, is observed to recruit the TLS (translocated in sarcoma) RNA-binding protein that represses transcription of the gene [143]. Therefore, expression of *CCND1* could be controlled by regulating its promoter associated lncRNA to impede downstream apoptotic signaling. 

#### 3.3.3. Inflammation

Inflammation is induced as an early event during cerebral ischemia. Cell death occurs almost immediately following occlusion of a cerebral vessel. This event, releases high-mobility group box 1 (HMGB1) and damage associated molecular patterns (DAMPs), which are well characterized in cerebral ischemia [214]. Binding of HMGB1 to toll-like receptors (TLRs), such as Toll-like receptor 4 (*TLR4*), activates astrocytes and microglia, to release cytokines and chemokines that potentiate an inflammatory response [215]. Subsequently, activated microglia generates reactive oxygen species (ROS) that contribute to neuronal cell death [216]. Cytokines such as Interleukin-1 beta (*IL1β*), Interleukin 6 (*IL-6*) and tumor necrosis factor alpha (*TNF-α*) [217,218,219], induce elevated expression of cell adhesion molecules (*E-selectins*, vascular cell adhesion molecule 1 (*VCAM-1*) and intracellular adhesion molecule 1 (*ICAM-1*)) in endothelial cells. These in turn recruit circulating immune cells (neutrophils, monocytes, T-cells) along with the endothelial cells [220]. Chemokines released from astrocytes also act as chemotactic cues for the immune cells to extravasate from the blood vessel and into the brain parenchyma [221]. The localization of immune cells in the brain aggravates ischemic injury with further cytokine secretion from the immune cells. Moreover, secretion of matrix metalloproteinases (MMPs) from neutrophils, results in blood brain barrier (BBB) disruption and formation of edema, exacerbating the effects of the injury [222].

miR-146a has been considered as a master regulator of inflammatory response. miR-146a/b are known to target interleukin-1 receptor-associated kinase 1 (*IRAK1*) and tumor necrosis factor (*TNF*) receptor associated factor protein 6 (*TRAF6*) mRNA, important molecules in signal transduction in TLR/IL1 receptor signaling [155]. Furthermore, miR-146a targets *TLR4*, a crucial molecule in mediating the early inflammatory response during ischemic injury [157]. 

miR-125b and miR-181c have been validated to target *TNF-α* that is increased upon ischemic injury [158,159]. Downregulation of *TNF-α* mRNA using miR-125b and miR-181c mimics may reduce inflammation during cerebral ischemia to aid in cell survival. miR-17 and miR-126 were reported to target *ICAM1* and *VCAM1* mRNA respectively [160,161]. These adhesion molecules are important for recruitment of immune cells into the brain parenchyma. miR-124 and -126 have been shown to target chemokine (C–C motif) ligand 2 (*CCL2*), a chemokine, which recruits monocytes into the brain parenchyma [223,224]. miR-17, miR-124 and miR-126 antagomirs can thus impede the recruitment of immune cells into the brain and thereby alleviate immune cell mediated injury. Hence, inflammatory response associated miRNAs may serve as potential neuroprotectants.

It is noteworthy that there are miRNAs associated with ischemia induced edema. miR-130a and miR-320a have been shown to repressor aquaporin 4 (*AQP4*) expression [22,52]. AQP4 is a bidirectional water conducting channel involved in early phase edema formation and edema clearance in the recovery phase. It was demonstrated that anti-miR-130a and -320a reduce infarct volume in rats subjected to middle cerebral artery occlusion (MCAo) by aiding in edema clearance.

LncRNA 17A is embedded in the human G-protein-coupled receptor 51 gene (*GPR51)* that codes for gamma-aminobutyric acid B receptor, 2 (GABAB R2) variant A and regulates alternative splicing of the gene [146]. Expression of 17A is increased upon inflammatory stimuli, driving the expression of GABAB R2 variant B, an alternative GABAB R2 protein isoform, devoid of transductional activity. This occurs alongside a dramatic downregulation of the canonical full-length GABAB R2 variant A, thereby abolishing GABAB R2 intracellular signaling and activation of K^+^ channels. These elevated levels of lnc 17A are shown to lead to an enhanced secretion of amyloid β peptide. 

#### 3.3.4. Oxidative Stress

Excessive production of reactive oxygen species (ROS) results in oxidative stress. During cerebral ischemia, influx of Ca^2+^, recruitment of immune cells and ischemic-reperfusion injuries leads to oxidative stress [225]. ROS causes DNA modification, protein denaturation, mitochondrial dysfunction, as well as lipid peroxidation, which results in membrane disruption [225], leading to neuronal cell death. Furthermore, the high lipid content of the brain makes it prone to lipid peroxidation that produces neurotoxic products, leading to oxidative stress [226,227]. In addition, the brain is enriched in iron which catalyzes the Fenton reaction to produce ROS. Anti-oxidant, superoxide dismutase 2 (*SOD2*) has been shown to be the target of miR-145 [152]. Antagonizing the expression of miR-145 was reported to reduce cerebral infarct size and protect neurons from oxidative stress. miR-101 targets cyclooxygenase 2 (*COX2*) mRNA, an enzyme involved in arachidonic acid metabolism, which generates ROS as a by-product [154]. Hence, introduction of pre-miR-101 can reduce ROS generation by inhibiting *COX2* expression. 

LncRNA MSUR1 (mutant SOD1-up-regulated RNA 1), has been shown to reduce free radical levels and oxidative damage resulting from mutant SOD1-mediated cell death [153]. Another lncRNA, growth arrested DNA-damage inducible gene 7 (Gadd7), is induced by lipotoxic stress in a ROS-dependent manner and is necessary for lipid- as well as general oxidative stress-mediated cell death. Knockdown of gadd7 has been shown to reverse this oxidative stress as well with reduced ROS production [155]. The data obtained thus far suggested the possibility of regulating these lncRNAs to serve as neuroprotectants against oxidative stress upon ischemic injury.

#### 3.3.5. Apoptosis

Apoptosis during cerebral ischemia is induced by internal signaling within the cell (intrinsic pathway) or by signal transduction from extracellular origin (extrinsic pathway) [23]. The intrinsic pathway is usually initiated by cues (associated with cellular damage) such as DNA modification, which activate p53 signaling; and mitochondrial dysfunction. p53 initiates the transcription of various apoptotic genes like B-cell lymphoma 2 (*Bcl-2*) and Bcl-2-associated X (*Bax*). Simultaneously, Bcl-2-like protein 11 (Bcl2l11), binds to other anti-apoptotic members of the Bcl2 family to induce apoptosis [228]. Furthermore, pro-apoptotic factors like Bax bind BH3-interacting domain death agonist (Bid) form pores on the mitochondria. This process promotes the release of factors that give rise to downstream formation of the apoptosome and mediates apoptosis through proteolytic cleavage of important downstream proteins [229]. 

The extrinsic pathway is induced by an external signal where ligands such as TNF-α and Fas ligand (FasL) bind to TNF-α receptor and Fas receptor (FasR) respectively [23]. Fas-Associated protein with Death Domain (FADD) is recruited to the receptor and forms the death-inducing signaling complex (DISC) together with procaspase 8 which subsequently converges with apoptotic signaling via the intrinsic pathway [229]. 

miR-15a, miR-29b, miR-497 were demonstrated to target members of the anti-apoptotic *Bcl-2* mRNA. miR-15a targets *Bcl-2* mRNA and miR-29b targets Bcl2-like protein 2 (*Bcl2l2*) while miR-497 targets both *Bcl-2* and *Bcl2l2* mRNAs [21,156,158]. Hence, miR-15a, miR-29b and miR-497 antagomirs can bring about potential neuroprotection by up-regulating anti-apoptotic proteins (Bcl-2, Bcl2l2). miR-21 prevents neurons from ischemic cell death by targeting FAS ligand (*FASLG*) [20]. Thus, treatment with pre-miR-21 can be neuroprotective during cerebral ischemia. In addition, miR-21 has been further demonstrated to protect neurons from microglial mediated neuronal cell death by targeting *FASLG* in microglial following ischemia [230]. 

miR-181 targets *GRP78* (heat shock 70kDa protein 5; glucose-regulated protein, 78kDa) during cerebral ischemia and the down-regulation of miR-181 was demonstrated to be neuroprotective [231]. Interestingly, inhibition of let-7f was shown to provide neuroprotection in middle-age female rats as let-7f targets components of the insulin-like growth factor 1 (IGF-1) signaling pathway [232].

The taurine up-regulated gene 1 (*TUG1*) codes for an lncRNA that is highly expressed in the cortex and is required for proper formation of photoreceptors in the developing rodent retina [233]. Downregulation of lncRNA TUG1 in developing retina leads to decreased retinal transcription factor expression and increased apoptosis. *TUG1* expression is activated by p53 upon DNA damage by repressing several cell cycle genes [157]. Expression of this ncRNA upon ischemic injury needs to be determined for its role in conferring cell death. 

Thus far, we have discussed the various ncRNAs, particularly miRNAs and lncRNAs, which are implicated in neuronal development, IP and major processes in the ischemic cascade. Amongst these, the regulatory functions of miRNAs have been the most extensively studied and well characterized. Although the studies on lncRNAs were mostly speculative, the preliminary results reported so far have highlighted their importance in the genome regulation with promising potential as key players in ischemia. Unfortunately, not much research has been done for piRNAs. In fact, none have been determined for their association with ischemia. Further in-depth studies are imperative to unveil the significance of these ncRNAs as potential targets for novel and/or alternative therapy in ischemic stroke. 

## 4. Future Directions for Therapy

Research into the roles of ncRNAs in ischemic brain injury is exponentially growing and the next approved therapy for ischemic stroke may just be targeting of ncRNAs. However, it should be noted that there are several benefits and drawbacks associated with the various subtypes of ncRNAs. In the case of miRNAs, there is the constant issue of off-target effects as they can potentially target hundreds of genes. Nonetheless, it is possible to utilize this multi-target characteristic to bring about a net neuroprotective effect. An example is that of miR-320a which targets multiple genes other than AQP4, in other pathways (inflammation, calcium signaling, cell cycle and apoptosis) associated with cerebral ischemia. This is responsible for an overall effect of reducing infarct volume in rats subjected to MCAo [22]. LncRNAs on the other hand may overcome this problem with increased specificity to their target gene and can be modulated through targeting by siRNAs.

One of the foremost questions for ncRNA therapy is the method of delivery and problem of crossing the blood brain barrier. The technology for ncRNA delivery is still in its infancy. The delivery of ncRNAs is mainly through viral vectors and nanoparticles or simply by using modified oligonucleotide (locked nucleic acid) backbone [234]. An intranasal delivery of ncRNAs can directly bypass the blood-brain barrier and serve as a non-invasive method. Recent developments have overcome this problem with the synthesis of exosomes that can cross the blood brain barrier to target neurons [235]. These exosomes are produced from mouse dendritic cells, which contain lysosomal-associated membrane protein 2B (Lamp2B) that binds to neuron specific peptides. Upon binding to neurons, the exosomes release siRNAs that were previously loaded by electroporation. Thus, this technology can be further developed to load ncRNAs for delivery into the ischemic brain. 

## 5. Open Questions in the Future

The potential use of the once considered “transcriptional noise” [3] as a therapeutic agent cannot be refuted given the exceptional interest and expanding achievements in this new field of research. Moreover, given their endogenous nature, the incidence of side effects of these ncRNAs as compared to synthetic drugs will also be significantly reduced. A mere two decades following the discovery of miRNAs by Lee *et al.* [236], it has led to several miRNAs being developed in the preclinical trials. miR-208 and miR-499 have undergone preclinical trial as antagonists for chronic heart failure [237]. Similarly, let-7 and miR-34 are currently in the preclinical phase for miRNA replacement therapy for cancer [238]. Adding to its feasibility, miR-122 antagomir is currently in phase II of clinical trials as a therapeutic for hepatitis C [239], the furthest development stage for any miRNA therapy to date. ncRNA treatment for ischemic brain injury is therefore plausible in the near future. 

We have identified several miRNAs and lncRNAs to be dysregulated upon ischemic injury. Of these, some ncRNAs exhibit potential as therapeutic targets. One of these is the BDNF-AS lncRNA. Administration of BDNF upon ischemic injury can effectively reduce cortical cell death and reduce infarct volume in *in vivo* stroke models [240,241]. However, these findings could not be effectively translated to clinics due to the inability of the BDNF protein to cross the blood-brain-barrier (BBB) in human subjects [242]. Hence, the neuroprotective role of BDNF can be exploited by using the more efficacious molecules such as siRNAs that are capable of crossing the BBB. Moreover, siRNAs have been successfully used against BDNF-AS to increase neuronal outgrowth *in vivo* [109]. It is therefore possible to extend this therapy to *in vivo* stroke models to target the BDNF-AS transcript, to stabilize the BDNF mRNA. In addition to targeting lncRNAs in neuronal development, ncRNAs identified in other ischemia induced detrimental processes also hold promise for therapy. MSUR1, 17A, Gadd7 and TUG1, induced by ischemia-related pathological processes (Table 2), could be modulated by siRNAs to verify their possible role as neuroprotectants.

Similarly, miRNA antagomirs or mimics that provide a net beneficial effect in ischemic brain injury such as anti-miR-320a [22], could be further explored as therapy. In-depth analyses on the potential use of pre-miR-124, pre-miR-9 (neurogenesis), pre-miR-146a (inflammation), pre-miR-21, anti-miR-497 (apoptosis) and anti-miR-15a (angiogenesis and apoptosis) for neuroprotective potential could serve as excellent starting points.

Further studies on these ncRNAs are thus necessary to understand the complex mechanisms and processes that are intricately regulated in the central nervous system. Ability to control these processes will pave a way for therapeutic intervention in ischemic injury.

## 6. Conclusion

Regarded as “junk” material at the start of the genome era, ncRNAs have now captured center stage being discovered as the crucial regulators of gene expression. The importance of ncRNAs in cell survival, death and disease are beginning to be unraveled. Though miRNAs have been studied quite rigorously and have found their way to therapeutic discoveries, knowledge of the functions of other ncRNAs is still in its infancy. However, with the recent trend of research shifting its focus towards elucidating the role of ncRNAs in pathological conditions, it is just a matter of time before ncRNA-based treatment for cerebral ischemia materializes. 

## References

[1] Dunham I., Kundaje A., Aldred S.F., Collins P.J., Davis C.A., Doyle F., Epstein C.B., Frietze S., Harrow J., Kaul R. (2012). An integrated encyclopedia of DNA elements in the human genome. Nature.

[2] Djebali S., Davis C.A., Merkel A., Dobin A., Lassmann T., Mortazavi A., Tanzer A., Lagarde J., Lin W., Schlesinger F. (2012). Landscape of transcription in human cells. Nature.

[3] Ponjavic J., Ponting C.P., Lunter G. (2007). Functionality or transcriptional noise? Evidence for selection within long noncoding RNAs. Genome Res..

[4] Qureshi I.A., Mehler M.F. (2010). The emerging role of epigenetics in stroke: II. RNA regulatory circuitry. Arch. Neurol..

[5] Ponting C.P., Oliver P.L., Reik W. (2009). Evolution and functions of long noncoding RNAs. Cell.

[6] Miska E.A., Alvarez-Saavedra E., Townsend M., Yoshii A., Sestan N., Rakic P., Constantine-Paton M., Horvitz H.R. (2004). Microarray analysis of microRNA expression in the developing mammalian brain. Genome Biol..

[7] Ng S.Y., Johnson R., Stanton L.W. (2012). Human long non-coding RNAs promote pluripotency and neuronal differentiation by association with chromatin modifiers and transcription factors. EMBO J..

[8] Lagos-Quintana M., Rauhut R., Yalcin A., Meyer J., Lendeckel W., Tuschl T. (2002). Identification of tissue-specific microRNAs from mouse. Curr. Biol..

[9] Cheng L.C., Pastrana E., Tavazoie M., Doetsch F. (2009). MiR-124 regulates adult neurogenesis in the subventricular zone stem cell niche. Nat. Neurosci..

[10] Mercer T.R., Qureshi I.A., Gokhan S., Dinger M.E., Li G., Mattick J.S., Mehler M.F. (2010). Long noncoding RNAs in neuronal-glial fate specification and oligodendrocyte lineage maturation. BMC Neurosci..

[11] Faghihi M.A., Modarresi F., Khalil A.M., Wood D.E., Sahagan B.G., Morgan T.E., Finch C.E., St Laurent G., Kenny P.J., Wahlestedt C. (2008). Expression of a noncoding RNA is elevated in Alzheimer’s disease and drives rapid feed-forward regulation of beta-secretase. Nat. Med..

[12] Bian S., Sun T. (2011). Functions of noncoding RNAs in neural development and neurological diseases. Mol. Neurobiol..

[13] Jeyaseelan K., Lim K.Y., Armugam A. (2008). MicroRNA expression in the blood and brain of rats subjected to transient focal ischemia by middle cerebral artery occlusion. Stroke.

[14] Dharap A., Nakka V.P., Vemuganti R. (2012). Effect of focal ischemia on long noncoding RNAs. Stroke.

[15] Roger V.L., Go A.S., Lloyd-Jones D.M., Benjamin E.J., Berry J.D., Borden W.B., Bravata D.M., Dai S., Ford E.S., Fox C.S. (2012). Heart disease and stroke statistics—2012 update: A report from the american heart association. Circulation.

[16] Tan J.R., Koo Y.X., Kaur P., Liu F., Armugam A., Wong P.T., Jeyaseelan K. (2011). MicroRNAs in stroke pathogenesis. Curr. Mol. Med..

[17] Jeyaseelan K., Lim K.Y., Armugam A. (2008). Neuroprotectants in stroke therapy. Expert Opin. Pharmacother..

[18] Akins P.T., Liu P.K., Hsu C.Y. (1996). Immediate early gene expression in response to cerebral ischemia. Friend or foe?. Stroke.

[19] Liu S., Levine S.R., Winn H.R. (2010). Targeting ischemic penumbra: Part I—From pathophysiology to therapeutic strategy. J. Exp. Stroke Transl. Med..

[20] Buller B., Liu X., Wang X., Zhang R.L., Zhang L., Hozeska-Solgot A., Chopp M., Zhang Z.G. (2010). MicroRNA-21 protects neurons from ischemic death. FEBS J..

[21] Yin K.J., Deng Z., Huang H., Hamblin M., Xie C., Zhang J., Chen Y.E. (2010). MiR-497 regulates neuronal death in mouse brain after transient focal cerebral ischemia. Neurobiol. Dis..

[22] Sepramaniam S., Armugam A., Lim K.Y., Karolina D.S., Swaminathan P., Tan J.R., Jeyaseelan K. (2010). MicroRNA 320a functions as a novel endogenous modulator of aquaporins 1 and 4 as well as a potential therapeutic target in cerebral ischemia. J. Biol. Chem..

[23] Broughton B.R., Reutens D.C., Sobey C.G. (2009). Apoptotic mechanisms after cerebral ischemia. Stroke.

[24] Nie L., Wu H.J., Hsu J.M., Chang S.S., Labaff A.M., Li C.W., Wang Y., Hsu J.L., Hung M.C. (2012). Long non-coding RNAs: Versatile master regulators of gene expression and crucial players in cancer. Am. J. Transl. Res..

[25] Bartel D.P. (2004). MicroRNAs: Genomics, biogenesis, mechanism, and function. Cell.

[26] Shi Y., Zhao X., Hsieh J., Wichterle H., Impey S., Banerjee S., Neveu P., Kosik K.S. (2010). MicroRNA regulation of neural stem cells and neurogenesis. J. Neurosci..

[27] Malone C.D., Hannon G.J. (2009). Small RNAs as guardians of the genome. Cell.

[28] Hartig J.V., Tomari Y., Forstemann K. (2007). piRNAs—The ancient hunters of genome invaders. Genes Dev..

[29] Thompson D.M., Parker R. (2009). Stressing out over tRNA cleavage. Cell.

[30] Cao F., Li X., Hiew S., Brady H., Liu Y., Dou Y. (2009). Dicer independent small RNAs associate with telomeric heterochromatin. RNA.

[31] Carone D.M., Longo M.S., Ferreri G.C., Hall L., Harris M., Shook N., Bulazel K.V., Carone B.R., Obergfell C., O’Neill M.J. (2009). A new class of retroviral and satellite encoded small RNAs emanates from mammalian centromeres. Chromosoma.

[32] Guttman M., Amit I., Garber M., French C., Lin M.F., Feldser D., Huarte M., Zuk O., Carey B.W., Cassady J.P. (2009). Chromatin signature reveals over a thousand highly conserved large non-coding RNAs in mammals. Nature.

[33] Louro R., Smirnova A.S., Verjovski-Almeida S. (2009). Long intronic noncoding RNA transcription: Expression noise or expression choice?. Genomics.

[34] Magistri M., Faghihi M.A., St Laurent G., Wahlestedt C. (2012). Regulation of chromatin structure by long noncoding RNAs: Focus on natural antisense transcripts. Trends Genet..

[35] Zheng D., Frankish A., Baertsch R., Kapranov P., Reymond A., Choo S.W., Lu Y., Denoeud F., Antonarakis S.E., Snyder M. (2007). Pseudogenes in the encode regions: Consensus annotation, analysis of transcription, and evolution. Genome Res..

[36] Zhang Z.D., Frankish A., Hunt T., Harrow J., Gerstein M. (2010). Identification and analysis of unitary pseudogenes: Historic and contemporary gene losses in humans and other primates. Genome Biol..

[37] Burzio V.A., Villota C., Villegas J., Landerer E., Boccardo E., Villa L.L., Martinez R., Lopez C., Gaete F., Toro V. (2009). Expression of a family of noncoding mitochondrial RNAs distinguishes normal from cancer cells. Proc. Natl. Acad. Sci. USA.

[38] Faulkner G.J., Kimura Y., Daub C.O., Wani S., Plessy C., Irvine K.M., Schroder K., Cloonan N., Steptoe A.L., Lassmann T. (2009). The regulated retrotransposon transcriptome of mammalian cells. Nat. Genet..

[39] Ferri F., Bouzinba-Segard H., Velasco G., Hube F., Francastel C. (2009). Non-coding murine centromeric transcripts associate with and potentiate aurora b kinase. Nucleic Acids Res..

[40] Taft R.J., Simons C., Nahkuri S., Oey H., Korbie D.J., Mercer T.R., Holst J., Ritchie W., Wong J.J., Rasko J.E. (2010). Nuclear-localized tiny RNAs are associated with transcription initiation and splice sites in metazoans. Nat. Struct. Mol. Biol..

[41] Preker P., Nielsen J., Kammler S., Lykke-Andersen S., Christensen M.S., Mapendano C.K., Schierup M.H., Jensen T.H. (2008). RNA exosome depletion reveals transcription upstream of active human promoters. Science.

[42] Kikuchi K., Fukuda M., Ito T., Inoue M., Yokoi T., Chiku S., Mitsuyama T., Asai K., Hirose T., Aizawa Y. (2009). Transcripts of unknown function in multiple-signaling pathways involved in human stem cell differentiation. Nucleic Acids Res..

[43] Kapranov P., Cheng J., Dike S., Nix D.A., Duttagupta R., Willingham A.T., Stadler P.F., Hertel J., Hackermuller J., Hofacker I.L. (2007). RNA maps reveal new RNA classes and a possible function for pervasive transcription. Science.

[44] Orom U.A., Derrien T., Beringer M., Gumireddy K., Gardini A., Bussotti G., Lai F., Zytnicki M., Notredame C., Huang Q. (2010). Long noncoding RNAs with enhancer-like function in human cells. Cell.

[45] Schratt G.M., Tuebing F., Nigh E.A., Kane C.G., Sabatini M.E., Kiebler M., Greenberg M.E. (2006). A brain-specific microRNA regulates dendritic spine development. Nature.

[46] Costa-Mattioli M., Sossin W.S., Klann E., Sonenberg N. (2009). Translational control of long-lasting synaptic plasticity and memory. Neuron.

[47] Lai X., Schmitz U., Gupta S.K., Bhattacharya A., Kunz M., Wolkenhauer O., Vera J. (2012). Computational analysis of target hub gene repression regulated by multiple and cooperative miRNAs. Nucleic Acids Res..

[48] Mukherji S., Ebert M.S., Zheng G.X.Y., Tsang J.S., Sharp P.A., van Oudenaarden A. (2011). MicroRNAs can generate thresholds in target gene expression. Nat. Genet..

[49] Krol J., Loedige I., Filipowicz W. (2010). The widespread regulation of microRNA biogenesis, function and decay. Nat. Rev. Genet..

[50] Li L.C., Okino S.T., Zhao H., Pookot D., Place R.F., Urakami S., Enokida H., Dahiya R. (2006). Small dsRNAs induce transcriptional activation in human cells. Proc. Natl. Acad. Sci. USA.

[51] Janowski B.A., Younger S.T., Hardy D.B., Ram R., Huffman K.E., Corey D.R. (2007). Activating gene expression in mammalian cells with promoter-targeted duplex RNAs. Nat. Chem. Biol..

[52] Sepramaniam S., Ying L.K., Armugam A., Wintour E.M., Jeyaseelan K. (2012). MicroRNA-130a represses transcriptional activity of aquaporin 4 mL promoter. J. Biol. Chem..

[53] Mercer T.R., Dinger M.E., Mattick J.S. (2009). Long non-coding RNAs: Insights into functions. Nat. Rev. Genet..

[54] Moran V.A., Perera R.J., Khalil A.M. (2012). Emerging functional and mechanistic paradigms of mammalian long non-coding RNAs. Nucleic Acids Res..

[55] Derrien T., Johnson R., Bussotti G., Tanzer A., Djebali S., Tilgner H., Guernec G., Martin D., Merkel A., Knowles D.G. (2012). The gencode v7 catalog of human long noncoding RNAs: Analysis of their gene structure, evolution, and expression. Genome Res..

[56] Wei W., Pelechano V., Jarvelin A.I., Steinmetz L.M. (2011). Functional consequences of bidirectional promoters. Trends Genet..

[57] Sone M., Hayashi T., Tarui H., Agata K., Takeichi M., Nakagawa S. (2007). The mRNA-like noncoding RNA gomafu constitutes a novel nuclear domain in a subset of neurons. J. Cell Sci..

[58] Feng J.C., Bi C.M., Clark B.S., Mady R., Shah P., Kohtz J.D. (2006). The Evf-2 noncoding RNA is transcribed from the Dlx-5/6 ultraconserved region and functions as a Dlx-2 transcriptional coactivator. Genes Dev..

[59] Rinn J.L., Kertesz M., Wang J.K., Squazzo S.L., Xu X., Brugmann S.A., Goodnough L.H., Helms J.A., Farnham P.J., Segal E. (2007). Functional demarcation of active and silent chromatin domains in human HOX loci by noncoding RNAs. Cell.

[60] Wilusz J.E., Sunwoo H., Spector D.L. (2009). Long noncoding RNAs: Functional surprises from the RNA world. Genes Dev..

[61] Siomi H., Siomi M.C. (2009). On the road to reading the RNA-interference code. Nature.

[62] Aravin A., Gaidatzis D., Pfeffer S., Lagos-Quintana M., Landgraf P., Iovino N., Morris P., Brownstein M.J., Kuramochi-Miyagawa S., Nakano T. (2006). A novel class of small RNAs bind to MILI protein in mouse testes. Nature.

[63] Girard A., Sachidanandam R., Hannon G.J., Carmell M.A. (2006). A germline-specific class of small RNAs binds mammalian Piwi proteins. Nature.

[64] Lau N.C., Seto A.G., Kim J., Kuramochi-Miyagawa S., Nakano T., Bartel D.P., Kingston R.E. (2006). Characterization of the piRNA complex from rat testes. Science.

[65] Aravin A.A., Hannon G.J., Brennecke J. (2007). The Piwi-piRNA pathway provides an adaptive defense in the transposon arms race. Science.

[66] Aravin A.A., Sachidanandam R., Bourc'his D., Schaefer C., Pezic D., Toth K.F., Bestor T., Hannon G.J. (2008). A piRNA pathway primed by individual transposons is linked to *de novo* DNA methylation in mice. Mol. Cell.

[67] Brennecke J., Aravin A.A., Stark A., Dus M., Kellis M., Sachidanandam R., Hannon G.J. (2007). Discrete small RNA-generating loci as master regulators of transposon activity in *Drosophila*. Cell.

[68] Siomi M.C., Sato K., Pezic D., Aravin A.A. (2011). Piwi-interacting small RNAs: The vanguard of genome defence. Nat. Rev. Mol. Cell Biol..

[69] Li C., Vagin V.V., Lee S., Xu J., Ma S., Xi H., Seitz H., Horwich M.D., Syrzycka M., Honda B.M. (2009). Collapse of germline piRNAs in the absence of argonaute3 reveals somatic piRNAs in flies. Cell.

[70] Malone C.D., Brennecke J., Dus M., Stark A., McCombie W.R., Sachidanandam R., Hannon G.J. (2009). Specialized piRNA pathways act in germline and somatic tissues of the *Drosophila* ovary. Cell.

[71] Gunawardane L.S., Saito K., Nishida K.M., Miyoshi K., Kawamura Y., Nagami T., Siomi H., Siomi M.C. (2007). A slicer-mediated mechanism for repeat-associated siRNA 5′ end formation in *Drosophila*. Science.

[72] Esteller M. (2011). Non-coding RNAs in human disease. Nat. Rev. Genet..

[73] Tonelli D.D., Pulvers J.N., Haffner C., Murchison E.P., Hannon G.J., Huttner W.B. (2008). MiRNAs are essential for survival and differentiation of newborn neurons but not for expansion of neural progenitors during early neurogenesis in the mouse embryonic neocortex. Development.

[74] Kawase-Koga Y., Otaegi G., Sun T. (2009). Different timings of dicer deletion affect neurogenesis and gliogenesis in the developing mouse central nervous system. Dev. Dyn..

[75] Davis T.H., Cuellar T.L., Koch S.M., Barker A.J., Harfe B.D., McManus M.T., Ullian E.M. (2008). Conditional loss of dicer disrupts cellular and tissue morphogenesis in the cortex and hippocampus. J. Neurosci..

[76] Lim L.P., Lau N.C., Garrett-Engele P., Grimson A., Schelter J.M., Castle J., Bartel D.P., Linsley P.S., Johnson J.M. (2005). Microarray analysis shows that some microRNAs downregulate large numbers of target mRNAs. Nature.

[77] Sanuki R., Onishi A., Koike C., Muramatsu R., Watanabe S., Muranishi Y., Irie S., Uneo S., Koyasu T., Matsui R. (2011). MiR-124a is required for hippocampal axogenesis and retinal cone survival through Lhx2 suppression. Nat. Neurosci..

[78] Maiorano N.A., Mallamaci A. (2009). Promotion of embryonic cortico-cerebral neuronogenesis by miR-124. Neural Dev..

[79] Conaco C., Otto S., Han J.J., Mandel G. (2006). Reciprocal actions of rest and a microRNA promote neuronal identity. Proc. Natl. Acad. Sci. USA.

[80] Visvanathan J., Lee S., Lee B., Lee J.W., Lee S.K. (2007). The microRNA miR-124 antagonizes the anti-neural REST/SCP1 pathway during embryonic CNS development. Genes Dev..

[81] Makeyev E.V., Zhang J., Carrasco M.A., Maniatis T. (2007). The microRNA miR-124 promotes neuronal differentiation by triggering brain-specific alternative pre-mRNA splicing. Mol. Cell.

[82] Liu X.S., Chopp M., Zhang R.L., Tao T., Wang X.L., Kassis H., Hozeska-Solgot A., Zhang L., Chen C., Zhang Z.G. (2011). MicroRNA profiling in subventricular zone after stroke: miR-124a regulates proliferation of neural progenitor cells through Notch signaling pathway. PLoS One.

[83] Weng H.C., Shen C.S., Hirokawa G., Ji X., Takahashi R., Shimada K., Kishimoto C., Iwai N. (2011). Plasma miR-124 as a biomarker for cerebral infarction. Biomed. Res..

[84] Leucht C., Stigloher C., Wizenmann A., Klafke R., Folchert A., Bally-Cuif L. (2008). MicroRNA-9 directs late organizer activity of the midbrain-hindbrain boundary. Nat. Neurosci..

[85] Deo M., Yu J.Y., Chung K.H., Tippens M., Turner D.L. (2006). Detection of mammalian microRNA expression by *in situ* hybridization with RNA oligonucleotides. Dev. Dyn..

[86] Packer A.N., Xing Y., Harper S.Q., Jones L., Davidson B.L. (2008). The bifunctional microRNA miR-9/miR-9* regulates REST and CoREST and is downregulated in Huntington’s disease. J. Neurosci..

[87] Zhao C., Sun G.Q., Li S.X., Shi Y.H. (2009). A feedback regulatory loop involving microRNA-9 and nuclear receptor TLX in neural stem cell fate determination. Nat. Struct. Mol. Biol..

[88] Delaloy C., Liu L., Lee J.A., Su H., Shen F., Yang G.Y., Young W.L., Ivey K.N., Gao F.B. (2010). MicroRNA-9 coordinates proliferation and migration of human embryonic stem cell-derived neural progenitors. Cell Stem Cell.

[89] Shibata M., Nakao H., Kiyonari H., Abe T., Aizawa S. (2011). MicroRNA-9 regulates neurogenesis in mouse telencephalon by targeting multiple transcription factors. J. Neurosci..

[90] Dajas-Bailador F., Bonev B., Garcez P., Stanley P., Guillemot F., Papalopulu N. (2012). MicroRNA-9 regulates axon extension and branching by targeting Map1b in mouse cortical neurons. Nat. Neurosci..

[91] Bruno L.G., Karam R., Huang L.L., Bhardwaj A., Lou C.H., Shum E.Y., Song H.W., Corbett M.A., Gifford W.D., Gecz J. (2011). Identification of a microRNA that activates gene expression by repressing nonsense-mediated RNA decay. Mol. Cell.

[92] Gaughwin P., Ciesla M., Yang H., Lim B., Brundin P. (2011). Stage-specific modulation of cortical neuronal development by Mmu-miR-134. Cereb. Cortex.

[93] Gao J., Wang W.Y., Mao Y.W., Graff J., Guan J.S., Pan L., Mak G., Kim D., Su S.C., Tsai L.H. (2010). A novel pathway regulates memory and plasticity via SIRT1 and miR-134. Nature.

[94] Fiore R., Khudayberdiev S., Christensen M., Siegel G., Flavell S.W., Kim T.K., Greenberg M.E., Schratt G. (2009). Mef2-mediated transcription of the miR379–410 cluster regulates activity-dependent dendritogenesis by fine-tuning Pumilio2 protein levels. EMBO J..

[95] Kole A.J., Swahari V., Hammond S.M., Deshmukh M. (2011). miR-29b is activated during neuronal maturation and targets BH3-only genes to restrict apoptosis. Genes Dev..

[96] Korneev S.A., Korneeva E.I., Lagarkova M.A., Kiselev S.L., Critchley G., O’Shea M. (2008). Novel noncoding antisense RNA transcribed from human anti-NOS2A locus is differentially regulated during neuronal differentiation of embryonic stem cells. RNA.

[97] Ling K.H., Hewitt C.A., Beissbarth T., Hyde L., Cheah P.S., Smyth G.K., Tan S.S., Hahn C.N., Thomas T., Thomas P.Q. (2011). Spatiotemporal regulation of multiple overlapping sense and novel natural antisense transcripts at the *Nrgn* and *Camk2n1* gene loci during mouse cerebral corticogenesis. Cereb. Cortex.

[98] Yoo A.S., Staahl B.T., Chen L., Crabtree G.R. (2009). MicroRNA-mediated switching of chromatin-remodelling complexes in neural development. Nature.

[99] Li K., Blum Y., Verma A., Liu Z., Pramanik K., Leigh N.R., Chun C.Z., Samant G.V., Zhao B., GaRNAas M.K. (2010). A noncoding antisense RNA in tie-1 locus regulates tie-1 function *in vivo*. Blood.

[100] Anguera M.C., Ma W.Y., Clift D., Namekawa S., Kelleher R.J., Lee J.T. (2011). Tsx produces a long noncoding RNA and has general functions in the germline, stem cells, and brain. PLoS Genet..

[101] BeRNArd D., Prasanth K.V., Tripathi V., Colasse S., Nakamura T., Xuan Z., Zhang M.Q., Sedel F., Jourdren L., Coulpier F. (2010). A long nuclear-retained non-coding RNA regulates synaptogenesis by modulating gene expression. EMBO J..

[102] Tripathi V., Ellis J.D., Shen Z., Song D.Y., Pan Q., Watt A.T., Freier S.M., Bennett C.F., Sharma A., Bubulya P.A. (2010). The nuclear-retained noncoding RNA MALAT1 regulates alternative splicing by modulating SR splicing factor phosphorylation. Mol. Cell.

[103] Li A.W., Seyoum G., Shiu R.P.C., Murphy P.R. (1996). Expression of the rat BFGF antisense RNA transcript is tissue-specific and developmentally regulated. Mol. Cell. Endorinol..

[104] Li A.W., Murphy P.R. (2000). Expression of alternatively spliced FGF-2 antisense RNA transcripts in the central nervous system: Regulation of FGF-2 mRNA translation. Mol. Cell. Endorinol..

[105] Zhang W., Chen Y., Liu P., Chen J., Song L., Tang Y., Wang Y., Liu J., Hu F.B., Hui R. (2012). Variants on chromosome 9p21.3 correlated with ANRIL expression contribute to stroke risk and recurrence in a large prospective stroke population. Stroke.

[106] Bond A.M., Vangompel M.J., Sametsky E.A., Clark M.F., Savage J.C., Disterhoft J.F., Kohtz J.D. (2009). Balanced gene regulation by an embryonic brain ncRNA is critical for adult hippocampal GABA circuitry. Nat. Neurosci..

[107] Amaral P.P., Neyt C., Wilkins S.J., Askarian-Amiri M.E., Sunkin S.M., Perkins A.C., Mattick J.S. (2009). Complex architecture and regulated expression of the Sox2ot locus during vertebrate development. RNA.

[108] Hawkins P.G., Morris K.V. (2010). Transcriptional regulation of Oct4 by a long non-coding RNA antisense to Oct4-pseudogene 5. Transcription.

[109] Modarresi F., Faghihi M.A., Lopez-Toledano M.A., Fatemi R.P., Magistri M., Brothers S.P., van der Brug M.P., Wahlestedt C. (2012). Inhibition of natural antisense transcripts *in vivo* results in gene-specific transcriptional upregulation. Nat. Biotechnol..

[110] Smrt R.D., Szulwach K.E., Pfeiffer R.L., Li X., Guo W., Pathania M., Teng Z.Q., Luo Y., Peng J., Bordey A. (2010). MicroRNA miR-137 regulates neuronal maturation by targeting ubiquitin ligase mind bomb-1. Stem Cells.

[111] Aranha M.M., Santos D.M., Sola S., Steer C.J., Rodrigues C.M.P. (2011). miR-34a regulates mouse neural stem cell differentiation. PLoS One.

[112] Wayman G.A., Davare M., Ando H., Fortin D., Varlamova O., Cheng H.Y.M., Marks D., Obrietan K., Soclerling T.R., Goodman R.H. (2008). An activity-regulated microRNA controls dendritic plasticity by down-regulating p250gap. Proc. Natl. Acad. Sci. USA.

[113] Luikart B.W., Bensen A.L., Washburn E.K., Perederiy J.V., Su K.G., Li Y., Kernie S.G., Parada L.F., Westbrook G.L. (2011). miR-132 mediates the integration of newborn neurons into the adult dentate gyrus. PLoS One.

[114] Aschrafi A., Schwechter A.D., Mameza M.G., Natera-Naranjo O., Gioio A.E., Kaplan B.B. (2008). MicroRNA-338 regulates local cytochrome *c* oxidase IV mRNA levels and oxidative phosphorylation in the axons of sympathetic neurons. J. Neurosci..

[115] Kye M.J., Liu T.L., Levy S.F., Xu N.L., Groves B.B., Bonneau R., Lao K.Q., Kosik K.S. (2007). Somatodendritic microRNAs identified by laser capture and multiplex RT-PCR. RNA.

[116] Edbauer D., Neilson J.R., Foster K.A., Wang C.F., Seeburg D.P., Batterton M.N., Tada T., Dolan B.M., Sharp P.A., Sheng M. (2010). Regulation of synaptic structure and function by FMRP-associated microRNAs miR-125b and miR-132. Neuron.

[117] Siegel G., Obernosterer G., Fiore R., Oehmen M., Bicker S., Christensen M., Khudayberdiev S., Leuschner P.F., Busch C.J.L., Kane C. (2009). A functional screen implicates microRNA-138-dependent regulation of the depalmitoylation enzyme APT1 in dendritic spine morphogenesis. Nat. Cell Biol..

[118] Banerjee S., Neveu P., Kosik K.S. (2009). A coordinated local translational control point at the synapse involving relief from silencing and MOV10 degradation. Neuron.

[119] Kocerha J., Faghihi M.A., Lopez-Toledano M.A., Huang J., Ramsey A.J., Caron M.G., Sales N., Willoughby D., Elmen J., Hansen H.F. (2009). MicroRNA-219 modulates NMDA receptor-mediated neurobehavioral dysfunction. Proc. Natl. Acad. Sci. USA.

[120] Abdelmohsen K., Hutchison E.R., Lee E.K., Kuwano Y., Kim M.M., Masuda K., Srikantan S., Subaran S.S., Marasa B.S., Mattson M.P. (2010). miR-375 inhibits differentiation of neurites by lowering HuD levels. Mol. Cell. Biol..

[121] Lee S.T., Chu K., Jung K.H., Yoon H.J., Jeon D., Kang K.M., Park K.H., Bae E.K., Kim M., Lee S.K. (2010). MicroRNAs induced during ischemic preconditioning. Stroke.

[122] Xu W.H., Yao X.Y., Yu H.J., Huang J.W., Cui L.Y. (2012). Downregulation of miR-199a may play a role in 3-nitropropionic acid induced ischemic tolerance in rat brain. Brain Res..

[123] Rane S., He M., Sayed D., Vashistha H., Malhotra A., Sadoshima J., Vatner D.E., Vatner S.F., Abdellatif M. (2009). Downregulation of miR-199a derepresses hypoxia-inducible factor-1alpha and Sirtuin 1 and recapitulates hypoxia preconditioning in cardiac myocytes. Circ. Res..

[124] Bertozzi D., Iurlaro R., Sordet O., Marinello J., Zaffaroni N., Capranico G. (2011). Characterization of novel antisense HIF-1alpha transcripts in human cancers. Cell Cycle.

[125] Taguchi A., Yanagisawa K., Tanaka M., Cao K., Matsuyama Y., Goto H., Takahashi T. (2008). Identification of hypoxia-inducible factor-1 alpha as a novel target for miR-17-92 microRNA cluster. Cancer Res..

[126] Thrash-Bingham C.A., Tartof K.D. (1999). aHIF: A natural antisense transcript overexpressed in human renal cancer and during hypoxia. J. Natl. Cancer Inst..

[127] Bruning U., Cerone L., Neufeld Z., Fitzpatrick S.F., Cheong A., Scholz C.C., Simpson D.A., Leonard M.O., Tambuwala M.M., Cummins E.P. (2011). MicroRNA-155 promotes resolution of hypoxia-inducible factor 1alpha activity during prolonged hypoxia. Mol. Cell. Biol..

[128] Bedo G., Vargas M., Ferreiro M.J., Chalar C., Agrati D. (2005). Characterization of hypoxia induced gene 1: Expression during rat central nervous system maturation and evidence of antisense RNA expression. Int. J. Dev. Biol..

[129] Song T., Zhang X., Wang C., Wu Y., Cai W., Gao J., Hong B. (2011). miR-138 suppresses expression of hypoxia-inducible factor 1alpha (HIF-1alpha) in clear cell renal cell carcinoma 786-O cells. Asian Pac. J. Cancer Prev..

[130] Pham N.V., Nguyen M.T., Hu J.F., Vu T.H., Hoffman A.R. (1998). Dissociation of IGF2 and H19 imprinting in human brain. Brain Res..

[131] Tsang W.P., Ng E.K., Ng S.S., Jin H., Yu J., Sung J.J., Kwok T.T. (2010). Oncofetal h19-derived miR-675 regulates tumor suppressor RB in human colorectal cancer. Carcinogenesis.

[132] Meng S., Cao J., Wang L., Zhou Q., Li Y., Shen C., Zhang X., Wang C. (2012). MicroRNA 107 partly inhibits endothelial progenitor cells differentiation via HIF-1beta. PLoS One.

[133] Poliseno L., Salmena L., Zhang J., Carver B., Haveman W.J., Pandolfi P.P. (2010). A coding-independent function of gene and pseudogene mRNAs regulates tumour biology. Nature.

[134] Lei Z., Li B., Yang Z., Fang H., Zhang G.M., Feng Z.H., Huang B. (2009). Regulation of HIF-1alpha and VEGF by miR-20b tunes tumor cells to adapt to the alteration of oxygen concentration. PLoS One.

[135] Sun C.Y., She X.M., Qin Y., Chu Z.B., Chen L., Ai L.S., Zhang L., Hu Y. (2012). miR-15a and miR-16 affect the angiogenesis of multiple myeloma by targeting VEGF. Carcinogenesis.

[136] Cha S.T., Chen P.S., Johansson G., Chu C.Y., Wang M.Y., Jeng Y.M., Yu S.L., Chen J.S., Chang K.J., Jee S.H. (2010). MicroRNA-519c suppresses hypoxia-inducible factor-1alpha expression and tumor angiogenesis. Cancer Res..

[137] Long J.Y., Wang Y., Wang W.J., Chang B.H.J., Danesh F.R. (2010). Identification of microRNA-93 as a novel regulator of vascular endothelial growth factor in hyperglycemic conditions. J. Biol. Chem..

[138] Sasahira T., Kurihara M., Bhawal U.K., Ueda N., Shimomoto T., Yamamoto K., Kirita T., Kuniyasu H. (2012). Downregulation of miR-126 induces angiogenesis and lymphangiogenesis by activation of VEGF-A in oral cancer. Br. J. Cancer.

[139] Roybal J.D., Zang Y., Ahn Y.H., Yang Y., Gibbons D.L., Baird B.N., Alvarez C., Thilaganathan N., Liu D.D., Saintigny P. (2011). miR-200 inhibits lung adenocarcinoma cell invasion and metastasis by targeting Flt1/VEGFR1. Mol. Cancer Res..

[140] Chen X., Gong J., Zeng H., Chen N., Huang R., Huang Y., Nie L., Xu M., Xia J., Zhao F. (2010). MicroRNA145 targets BNIP3 and suppresses prostate cancer progression. Cancer Res..

[141] Zhang C.Z., Zhang J.X., Zhang A.L., Shi Z.D., Han L., Jia Z.F., Yang W.D., Wang G.X., Jiang T., You Y.P. (2010). miR-221 and miR-222 target puma to induce cell survival in glioblastoma. Mol. Cancer.

[142] Harraz M.M., Eacker S.M., Wang X., Dawson T.M., Dawson V.L. (2012). MicroRNA-223 is neuroprotective by targeting glutamate receptors. Proc. Natl. Acad. Sci. USA.

[143] Wang X., Arai S., Song X., Reichart D., Du K., Pascual G., Tempst P., Rosenfeld M.G., Glass C.K., Kurokawa R. (2008). Induced ncRNAs allosterically modify RNA-binding proteins *in cis* to inhibit transcription. Nature.

[144] Saba R., Storchel P.H., Aksoy-Aksel A., Kepura F., Lippi G., Plant T.D., Schratt G.M. (2012). Dopamine-regulated microRNA miR-181a controls GluA2 surface expression in hippocampal neurons. Mol. Cell. Biol..

[145] Taganov K.D., Boldin M.P., Chang K.J., Baltimore D. (2006). NF-kappaB-dependent induction of microRNA miR-146, an inhibitor targeted to signaling proteins of innate immune responses. Proc. Natl. Acad. Sci. USA.

[146] Massone S., Vassallo I., Fiorino G., Castelnuovo M., Barbieri F., Borghi R., Tabaton M., Robello M., Gatta E., Russo C. (2011). 17a, a novel non-coding RNA, regulates GABA B alternative splicing and signaling in response to inflammatory stimuli and in Alzheimer disease. Neurobiol. Dis..

[147] Yang K., He Y.S., Wang X.Q., Lu L., Chen Q.J., Liu J., Sun Z., Shen W.F. (2011). MiR-146a inhibits oxidized low-density lipoprotein-induced lipid accumulation and inflammatory response via targeting toll-like receptor 4. FEBS Lett..

[148] Zhang L., Dong L.Y., Li Y.J., Hong Z., Wei W.S. (2012). The microRNA miR-181c controls microglia-mediated neuronal apoptosis by suppressing tumor necrosis factor. J. Neuroinflammation.

[149] Tili E., Michaille J.J., Cimino A., Costinean S., Dumitru C.D., Adair B., Fabbri M., Alder H., Liu C.G., Calin G.A. (2007). Modulation of miR-155 and miR-125b levels following lipopolysaccharide/TNF-alpha stimulation and their possible roles in regulating the response to endotoxin shock. J. Immunol..

[150] Suarez Y., Wang C., Manes T.D., Pober J.S. (2010). Cutting edge: TNF-induced microRNAs regulate TNF-induced expression of E-selectin and intercellular adhesion molecule-1 on human endothelial cells: Feedback control of inflammation. J. Immunol..

[151] Harris T.A., Yamakuchi M., Ferlito M., Mendell J.T., Lowenstein C.J. (2008). MicroRNA-126 regulates endothelial expression of vascular cell adhesion molecule 1. Proc. Natl. Acad. Sci. USA.

[152] Dharap A., Bowen K., Place R., Li L.C., Vemuganti R. (2009). Transient focal ischemia induces extensive temporal changes in rat cerebral microRNAome. J. Cereb. Blood Flow Metab..

[153] Chang Y.M., Stockinger M.P., Tashiro H., Lin C.L.G. (2008). A novel noncoding RNA rescues mutant SOD1-mediated cell death. FASEB J..

[154] Strillacci A., Griffoni C., Sansone P., Paterini P., Piazzi G., Lazzarini G., Spisni E., Pantaleo M.A., Biasco G., Tomasi V. (2009). MiR-101 downregulation is involved in cyclooxygenase-2 overexpression in human colon cancer cells. Exp. Cell Res..

[155] Brookheart R.T., Michel C.I., Listenberger L.L., Ory D.S., Schaffer J.E. (2009). The non-coding RNA gadd7 is a regulator of lipid-induced oxidative and endoplasmic reticulum stress. J. Biol. Chem..

[156] Yin K.J., Deng Z., Hamblin M., Xiang Y., Huang H., Zhang J., Jiang X., Wang Y., Chen Y.E. (2010). Peroxisome proliferator-activated receptor delta regulation of miR-15a in ischemia-induced cerebral vascular endothelial injury. J. Neurosci..

[157] Khalil A.M., Guttman M., Huarte M., Garber M., Raj A., Rivea Morales D., Thomas K., Presser A., Bernstein B.E., van Oudenaarden A. (2009). Many human large intergenic noncoding RNAs associate with chromatin-modifying complexes and affect gene expression. Proc. Natl. Acad. Sci. USA.

[158] Shi G., Liu Y., Liu T., Yan W., Liu X., Wang Y., Shi J., Jia L. (2012). Upregulated miR-29b promotes neuronal cell death by inhibiting Bcl2L2 after ischemic brain injury. Exp. Brain Res..

[159] Mercer T.R., Dinger M.E., Sunkin S.M., Mehler M.F., Mattick J.S. (2008). Specific expression of long noncoding RNAs in the mouse brain. Proc. Natl. Acad. Sci. USA.

[160] Zhu D.Y., Liu S.H., Sun H.S., Lu Y.M. (2003). Expression of inducible nitric oxide synthase after focal cerebral ischemia stimulates neurogenesis in the adult rodent dentate gyrus. J. Neurosci..

[161] Li L., Li Y., Ji X., Zhang B., Wei H., Luo Y. (2008). The effects of retinoic acid on the expression of neurogranin after experimental cerebral ischemia. Brain Res..

[162] Ryge J., Winther O., Wienecke J., Sandelin A., Westerdahl A.C., Hultborn H., Kiehn O. (2010). Transcriptional regulation of gene expression clusters in motor neurons following spinal cord injury. BMC Genomics.

[163] Lin T.N., Te J., Lee M., Sun G.Y., Hsu C.Y. (1997). Induction of basic fibroblast growth factor (BFGF) expression following focal cerebral ischemia. Brain Res. Mol. Brain Res..

[164] Ling K.H., Hewitt C.A., Beissbarth T., Hyde L., Banerjee K., Cheah P.S., Cannon P.Z., Hahn C.N., Thomas P.Q., Smyth G.K. (2009). Molecular networks involved in mouse cerebral corticogenesis and spatio-temporal regulation of Sox4 and Sox11 novel antisense transcripts revealed by transcriptome profiling. Genome Biol..

[165] Komine Y., Nakamura K., Katsuki M., Yamamori T. (2006). Novel transcription factor zfh-5 is negatively regulated by its own antisense RNA in mouse brain. Mol. Cell. Neurosci..

[166] Kerr N., Pintzas A., Holmes F., Hobson S.A., Pope R., Wallace M., Wasylyk C., Wasylyk B., Wynick D. (2010). The expression of ELK transcription factors in adult DRG: Novel isoforms, antisense transcripts and upregulation by nerve damage. Mol. Cell. Neurosci..

[167] Mohamed J.S., Gaughwin P.M., Lim B., Robson P., Lipovich L. (2010). Conserved long noncoding RNAs transcriptionally regulated by Oct4 and Nanog modulate pluripotency in mouse embryonic stem cells. RNA.

[168] Rajasethupathy P., Antonov I., Sheridan R., Frey S., Sander C., Tuschl T., Kandel E.R. (2012). A role for neuronal piRNAs in the epigenetic control of memory-related synaptic plasticity. Cell.

[169] Murry C.E., Jennings R.B., Reimer K.A. (1986). Preconditioning with ischemia—A delay of lethal cell injury in ischemic myocardium. Circulation.

[170] Kitagawa K., Matsumoto M., Kuwabara K., Tagaya M., Ohtsuki T., Hata R., Ueda H., Handa N., Kimura K., Kamada T. (1991). Ischemic tolerance phenomenon detected in various brain-regions. Brain Res..

[171] DiRNAgl U., Simon R.P., Hallenbeck J.M. (2003). Ischemic tolerance and endogenous neuroprotection. Trends Neurosci..

[172] Murphy E., Steenbergen C. (2008). Mechanisms underlying acute protection from cardiac ischemia-reperfusion injury. Physiol. Rev..

[173] Tejero-Taldo M.I., Gursoy E., Zhao T.C., Kukreja R.C. (2002). Alpha-adrenergic receptor stimulation produces late preconditioning through inducible nitric oxide synthase in mouse heart. J. Mol. Cell. Cardiol..

[174] Hampton C.R., Shimamoto A., Rothnie C.L., Griscavage-Ennis J., Chong A., Dix D.J., Verrier E.D., Pohlman T.H. (2003). HSP70.1 and -70.3 are required for late-phase protection induced by ischemic preconditioning of mouse hearts. Am. J. Physiol. Heart Circ. Physiol..

[175] Kaneko T., Yokoyama K., Makita K. (2005). Late preconditioning with isoflurane in cultured rat cortical neurones. Br. J. Anaesth..

[176] Dharap A., Vemuganti R. (2010). Ischemic pre-conditioning alters cerebral microRNAs that are upstream to neuroprotective signaling pathways. J. Neurochem..

[177] Lusardi T.A., Farr C.D., Faulkner C.L., Pignataro G., Yang T., Lan J.Q., Simon R.P., Saugstad J.A. (2010). Ischemic preconditioning regulates expression of microRNAs and a predicted target, mecp2, in mouse cortex. J. Cereb. Blood Flow Metab..

[178] Mehta S.L., Manhas N., Raghubir R. (2007). Molecular targets in cerebral ischemia for developing novel therapeutics. Brain Res. Rev..

[179] Tan K.S., Armugam A., Sepramaniam S., Lim K.Y., Setyowati K.D., Wang C.W., Jeyaseelan K. (2009). Expression profile of microRNAs in young stroke patients. PLoS One.

[180] Gupta R.A., Shah N., Wang K.C., Kim J., Horlings H.M., Wong D.J., Tsai M.C., Hung T., Argani P., Rinn J.L. (2010). Long non-coding RNA hotair reprograms chromatin state to promote cancer metastasis. Nature.

[181] Huarte M., Guttman M., Feldser D., Garber M., Koziol M.J., Kenzelmann-Broz D., Khalil A.M., Zuk O., Amit I., Rabani M. (2010). A large intergenic noncoding RNA induced by p53 mediates global gene repression in the p53 response. Cell.

[182] Kalkkila J.P., Sharp F.R., Karkkainen I., Reilly M., Lu A.G., Solway K., Murrel M., Honkaniemi J. (2004). Cloning and expression of short interspersed elements B1 and B2 in ischemic brain. Eur. J. Neurosci..

[183] Dharap A., Nakka V.P., Vemuganti R. (2011). Altered expression of piwi RNA in the rat brain after transient focal ischemia. Stroke.

[184] Sharma A.K., Nelson M.C., Brandt J.E., Wessman M., Mahmud N., Weller K.P., Hoffman R. (2001). Human CD34(+) stem cells express the hiwi gene, a human homologue of the *Drosophila* gene *piwi*. Blood.

[185] Semenza G.L. (2000). HIF-1: Mediator of physiological and pathophysiological responses to hypoxia. J. Appl. Physiol..

[186] Pugh C.W., Ratcliffe P.J. (2003). Regulation of angiogenesis by hypoxia: Role of the HIF system. Nat. Med..

[187] Bunn H.F., Gu J., Huang L.E., Park J.W., Zhu H. (1998). Erythropoietin: A model system for studying oxygen-dependent gene regulation. J. Exp. Biol..

[188] Cunningham L.A., Candelario K., Li L. (2012). Roles for HIF-1alpha in neural stem cell function and the regenerative response to stroke. Behav. Brain Res..

[189] Bruick R.K. (2003). Oxygen sensing in the hypoxic response pathway: Regulation of the hypoxia-inducible transcription factor. Genes Dev..

[190] Wiener C.M., Booth G., Semenza G.L. (1996). *In vivo* expression of mRNAs encoding hypoxia-inducible factor 1. Biochem. Biophys. Res. Commun..

[191] Bergeron M., Yu A.Y., Solway K.E., Semenza G.L., Sharp F.R. (1999). Induction of hypoxia-inducible factor-1 (HIF-1) and its target genes following focal ischaemia in rat brain. Eur. J. Neurosci..

[192] Yeh S.H., Ou L.C., Gean P.W., Hung J.J., Chang W.C. (2011). Selective inhibition of early-but not late-expressed HIF-1 alpha is neuroprotective in rats after focal ischemic brain damage. Brain Pathol..

[193] Ivan M., Harris A.L., Martelli F., Kulshreshtha R. (2008). Hypoxia response and microRNAs: No longer two separate worlds. J. Cell. Mol. Med..

[194] Pulkkinen K., Malm T., Turunen M., Koistinaho J., Yla-Herttuala S. (2008). Hypoxia induces microRNA miR-210 *in vitro* and *in vivo* ephrin-a3 and neuronal pentraxin 1 are potentially regulated by miR-210. FEBS Lett..

[195] Lou Y.L., Guo F., Liu F., Gao F.L., Zhang P.Q., Niu X., Guo S.C., Yin J.H., Wang Y., Deng Z.F. (2012). MiR-210 activates notch signaling pathway in angiogenesis induced by cerebral ischemia. Mol. Cell. Biochem..

[196] Zeng L., Liu J., Wang Y., Wang L., Weng S., Tang Y., Zheng C., Cheng Q., Chen S., Yang G.Y. (2011). MicroRNA-210 as a novel blood biomarker in acute cerebral ischemia. Front. Biosci. (Elite Ed.).

[197] Rossignol F., de Laplanche E., Mounier R., Bonnefont J., Cayre A., Godinot C., Simonnet H., Clottes E. (2004). Natural antisense transcripts of HIF-1alpha are conserved in rodents. Gene.

[198] Jin K., Mao X.O., Eshoo M.W., del Rio G., Rao R., Chen D., Simon R.P., Greenberg D.A. (2002). cDNA microarray analysis of changes in gene expression induced by neuronal hypoxia *in vitro*. Neurochem. Res..

[199] Shi G.D., OuYang Y.P., Shi J.G., Liu Y., Yuan W., Jia L.S. (2011). PTEN deletion prevents ischemic brain injury by activating the mTOR signaling pathway. Biochem. Biophys. Res. Commun..

[200] Cai Q.Y., Chen X.S., Zhong S.C., Luo X., Yao Z.X. (2009). Differential expression of PTEN in normal adult rat brain and upregulation of PTEN and *p*-Akt in the ischemic cerebral cortex. Anat. Rec. (Hoboken).

[201] Zundel W., Schindler C., Haas-Kogan D., Koong A., Kaper F., Chen E., Gottschalk A.R., Ryan H.E., Johnson R.S., Jefferson A.B. (2000). Loss of PTEN facilitates HIF-1-mediated gene expression. Genes Dev..

[202] Ly J.V., Zavala J.A., Donnan G.A. (2006). Neuroprotection and thrombolysis: Combination therapy in acute ischaemic stroke. Expert Opin. Pharmaco..

[203] Pizzi M., Fallacara C., Arrighi V., Memo M., Spano P. (1993). Attenuation of excitatory amino-acid toxicity by metabotropic glutamate-receptor agonists and aniracetam in primary cultures of cerebellar granule cells. J. Neurochem..

[204] Mosbacher J., Schoepfer R., Monyer H., BuRNAshev N., Seeburg P.H., Ruppersberg J.P. (1994). A molecular determinant for submillisecond desensitization in glutamate receptors. Science.

[205] Moriyoshi K., Masu M., Ishii T., Shigemoto R., Mizuno N., Nakanishi S. (1991). Molecular-cloning and characterization of the rat NMDA receptor. Nature.

[206] Budd S.L. (1998). Mechanisms of neuronal damage in brain hypoxia/ischemia: Focus on the role of mitochondrial calcium accumulation. Pharmacol. Ther..

[207] Wang Y., Qin Z.H. (2010). Molecular and cellular mechanisms of excitotoxic neuronal death. Apoptosis.

[208] Yamauchi M., Omote K., Ninomiya T. (1998). Direct evidence for the role of nitric oxide on the glutamate-induced neuronal death in cultured cortical neurons. Brain Res..

[209] Lazarewicz J.W., Wroblewski J.T., Costa E. (1990). *N*-methyl-d-aspartate-sensitive glutamate receptors induce calcium-mediated arachidonic-acid release in primary cultures of cerebellar granule cells. J. Neurochem..

[210] Kokaia Z., Zhao Q., Kokaia M., Elmer E., Metsis M., Smith M.L., Siesjo B.K., Lindvall O. (1995). Regulation of brain-derived neurotrophic factor gene expression after transient middle cerebral artery occlusion with and without brain damage. Exp. Neurol..

[211] Kawashima H., Numakawa T., Kumamaru E., Adachi N., Mizuno H., Ninomiya M., Kunugi H., Hashido K. (2010). Glucocorticoid attenuates brain-derived neurotrophic factor-dependent upregulation of glutamate receptors via the suppression of microRNA-132 expression. Neuroscience.

[212] Rashidian J., Iyirhiaro G., Aleyasin H., Rios M., Vincent I., Callaghan S., Bland R.J., Slack R.S., During M.J., Park D.S. (2005). Multiple cyclin-dependent kinases signals are critical mediators of ischemia/hypoxic neuronal death *in vitro* and *in vivo*. Proc. Natl. Acad. Sci. USA.

[213] Efthimiadi L., Farso M., Quirion R., Krantic S. (2012). Cyclin D1 induction preceding neuronal death via the excitotoxic NMDA pathway involves selective stimulation of extrasynaptic NMDA receptors and JNK pathway. Neurodegener. Dis..

[214] Hayakawa K., Qiu J.H., Lo E.H. (2010). Biphasic actions of HMGB1 signaling in inflammation and recovery after stroke. Ann. N. Y. Acad. Sci..

[215] Wang Q., Tang X.N., Yenari M.A. (2007). The inflammatory response in stroke. J. Neuroimmunol..

[216] Yenari M.A., Kauppinen T.M., Swanson R.A. (2010). Microglial activation in stroke: Therapeutic targets. Neurotherapeutics.

[217] Caso J.R., Moro M.A., Lorenzo P., Lizasoain I., Leza J.C. (2007). Involvement of IL-1 beta in acute stress-induced worsening of cerebral ischaemia in rats. Eur. Neuropsychopharm..

[218] Clark W.M., Rinker L.G., Lessov N.S., Hazel K.A., Eckenstein F.P. (1999). Time course of IL-6 expression in experimental CNS ischemia. Neurology.

[219] Liu T., Clark R.K., Mcdonnell P.C., Young P.R., White R.F., Barone F.C., Feuerstein G.Z. (1994). Tumor-necrosis-factor-alpha expression in ischemic neurons. Stroke.

[220] Yilmaz G., Granger D.N. (2008). Cell adhesion molecules and ischemic stroke. Neurol. Res..

[221] Mirabelli-Badenier M., Braunersreuther V., Viviani G.L., Dallegri F., Quercioli A., Veneselli E., Mach F., Montecucco F. (2011). CC and CXC chemokines are pivotal mediators of cerebral injury in ischaemic stroke. Thromb. Haemost..

[222] Jin R., Yang G.J., Li G.H. (2010). Molecular insights and therapeutic targets for blood-brain barrier disruption in ischemic stroke: Critical role of matrix metalloproteinases and tissue-type plasminogen activator. Neurobiol. Dis..

[223] Kawano S., Nakamachi Y. (2011). miR-124a as a key regulator of proliferation and MCP-1 secretion in synoviocytes from patients with rheumatoid arthritis. Ann. Rheum. Dis..

[224] Arner E., Mejhert N., Kulyte A., Balwierz P.J., Pachkov M., Cormont M., Lorente-Cebrian S., Ehrlund A., Laurencikiene J., Heden P. (2012). Adipose tissue microRNAs as regulators of CCL2 production in human obesity. Diabetes.

[225] Allen C.L., Bayraktutan U. (2009). Oxidative stress and its role in the pathogenesis of ischaemic stroke. Int. J. Stroke.

[226] Saeed S.A., Shad K.F., Saleem T., Javed F., Khan M.U. (2007). Some new prospects in the understanding of the molecular basis of the pathogenesis of stroke. Exp. Brain Res..

[227] McCracken E., Valeriani V., Simpson C., Jover T., McCulloch J., Dewar D. (2000). The lipid peroxidation by-product 4-hydroxynonenal is toxic to axons and oligodendrocytes. J. Cereb. Blood Flow Metab..

[228] Culmsee C., Mattson M.P. (2005). P53 in neuronal apoptosis. Biochem. Biophys. Res. Commun..

[229] Love S. (2003). Apoptosis and brain ischaemia. Prog. Neuropsychopharmacol. Biol. Psychiatry.

[230] Zhang L., Dong L.Y., Li Y.J., Hong Z., Wei W.S. (2012). MiR-21 represses FasL in microglia and protects against microglia-mediated neuronal cell death following hypoxia/ischemia. Glia.

[231] Ouyang Y.B., Lu Y., Yue S., Xu L.J., Xiong X.X., White R.E., Sun X., Giffard R.G. (2012). miR-181 regulates GRP78 and influences outcome from cerebral ischemia *in vitro* and *in vivo*. Neurobiol. Dis..

[232] Selvamani A., Sathyan P., Miranda R.C., Sohrabji F. (2012). An antagomir to microRNA Let7f promotes neuroprotection in an ischemic stroke model. PLoS One.

[233] Young T.L., Matsuda T., Cepko C.L. (2005). The noncoding RNA taurine upregulated gene 1 is required for differentiation of the murine retina. Curr. Biol..

[234] Pastori C., Wahlestedt C. (2012). Involvement of long noncoding RNAs in diseases affecting the central nervous system. RNA Biol..

[235] Alvarez-Erviti L., Seow Y., Yin H., Betts C., Lakhal S., Wood M.J. (2011). Delivery of siRNA to the mouse brain by systemic injection of targeted exosomes. Nat. Biotechnol..

[236] Lee R.C., Feinbaum R.L., Ambros V. (1993). The *C. elegans* heterochronic gene lin-4 encodes small RNAs with antisense complementarity to lin-14. Cell.

[237] Van Rooij E., Sutherland L.B., Qi X., Richardson J.A., Hill J., Olson E.N. (2007). Control of stress-dependent cardiac growth and gene expression by a microRNA. Science.

[238] Wiggins J.F., Ruffino L., Kelnar K., Omotola M., Patrawala L., Brown D., Bader A.G. (2010). Development of a lung cancer therapeutic based on the tumor suppressor microRNA-34. Cancer Res..

[239] Lanford R.E., Hildebrandt-Eriksen E.S., Petri A., Persson R., Lindow M., Munk M.E., Kauppinen S., Orum H. (2010). Therapeutic silencing of microRNA-122 in primates with chronic hepatitis c virus infection. Science.

[240] Schabitz W.R., Sommer C., Zoder W., Kiessling M., Schwaninger M., Schwab S. (2000). Intravenous brain-derived neurotrophic factor reduces infarct size and counterregulates bax and bcl-2 expression after temporary focal cerebral ischemia. Stroke.

[241] Ferrer I., Krupinski J., Goutan E., Marti E., Ambrosio S., Arenas E. (2001). Brain-derived neurotrophic factor reduces cortical cell death by ischemia after middle cerebral artery occlusion in the rat. Acta Neuropathol..

[242] Wu D. (2005). Neuroprotection in experimental stroke with targeted neurotrophins. NeuroRx.

